# Human 8-oxoguanine glycosylase OGG1 binds nucleosome at the dsDNA ends and the super-helical locations

**DOI:** 10.1038/s42003-024-06919-7

**Published:** 2024-09-28

**Authors:** Qinglong You, Xiang Feng, Yi Cai, Stephen B. Baylin, Huilin Li

**Affiliations:** 1 Department of Structural Biology, Van Andel Institute, Grand Rapids, MI USA; 2 Department of Oncology, Sidney Kimmel Comprehensive Cancer Center at Johns Hopkins, The Johns Hopkins University School of Medicine, Baltimore, MD 21287 USA; 3 Department of Epigenetics, Van Andel Institute, Grand Rapids, MI USA

**Keywords:** Cryoelectron microscopy, Base excision repair

## Abstract

The human glycosylase OGG1 extrudes and excises the oxidized DNA base 8-oxoguanine (8-oxoG) to initiate base excision repair and plays important roles in many pathological conditions such as cancer, inflammation, and neurodegenerative diseases. Previous structural studies have used a truncated protein and short linear DNA, so it has been unclear how full-length OGG1 operates on longer DNA or on nucleosomes. Here we report cryo-EM structures of human OGG1 bound to a 35-bp long DNA containing an 8-oxoG within an unmethylated Cp-8-oxoG dinucleotide as well as to a nucleosome with an 8-oxoG at super-helical location (SHL)-5. The 8-oxoG in the linear DNA is flipped out by OGG1, consistent with previous crystallographic findings with a 15-bp DNA. OGG1 preferentially binds near dsDNA ends at the nucleosomal entry/exit sites. Such preference may underlie the enzyme’s function in DNA double-strand break repair. Unexpectedly, we find that OGG1 bends the nucleosomal entry DNA, flips an undamaged guanine, and binds to internal nucleosomal DNA sites such as SHL-5 and SHL+6. We suggest that the DNA base search mechanism by OGG1 may be chromatin context-dependent and that OGG1 may partner with chromatin remodelers to excise 8-oxoG at the nucleosomal internal sites.

## Abstract (web summary)

Cryo-EM reveals that the human OGG1 prefers to bind at the nucleosomal super-helical locations as well as the DNA entry or exit site of mono nucleosome in vitro, which resembles a double-strand DNA break.

## Introduction

DNA is constantly damaged by environmental and endogenous factors^1^. Cells have evolved multiple DNA repair pathways, including base excision repair (BER), nucleotide excision repair, mismatch repair, homologous recombination, and non-homologous end joining to maintain genome integrity^2^. Exogenous DNA damaging factors include UV, ionizing radiation, alkylating, and crosslinking agents, and the main endogenous oxidative DNA damage factor is reactive oxygen species^3,4^. The most predominant oxidative damage to DNA is the oxidation of guanine (G) into 7,8-dihydro-8-oxoguanine (8-oxoG)^5–7^. 8-oxoG can base pair with adenine, leading to a G·C to T·A transversion via DNA replication^4,8–11^. Mammalian 8-oxoguanine glycosylase 1 (OGG1) and its bacterial homolog MutM excises 8-oxoG and initiates BER^10,12–16^. OGG1 is an important player in gene expression, cancer, inflammation, neurodegenerative diseases, and many pathological conditions^4,7,17–20^. In this regard for cancer, our group has found that OGG1 interaction with the ROS-damaged base 8-oxoG is the upstream event that leads to the transcriptional repression NURD complex binding to the gene start sites in the CpG islands, and the NURD interaction triggers cancer-specific DNA hypermethylation associated with abnormal silencing of tumor suppressor genes (TSG’s) and abnormalities of immune function^20^. Thus, OGG1 inhibitors are being developed as anti-cancer and anti-inflammatory agents^21–23^. Further, OGG1 activators have the potential to treat Alzheimer’s disease and obesity^24–29^.

Humans express a nucleus alpha splice form of OGG1 with 345 amino acids and a mitochondrial beta splice form with 424 amino acids^30,31^. These two variants share the first 316 amino acids and differ at the C-terminus that contains the signal sequences. The structure and function of human OGG1, especially the nuclear isoform OGG1-1a have been extensively studied^7,32–35^. OGG1 engages DNA in three steps, as revealed by the crystal structures of the enzyme-DNA complexes^34,36–38^: (1) interrogation complex in which OGG1 is at a fully intrahelical G:C base pair site; (2) encounter complex in which OGG1 encounters an 8-oxoG:C base pair that is sequence-matched and fully intrahelical site; and (3) lesion recognition complex in which 8-oxoG is extrahelical (extruded from the DNA helix), flipped, and inserts into the lesion recognition pocket of the enzyme. The interrogation and encounter complexes were captured by intermolecular disulfide crosslinking^36,37^. DNA is virtually unbent in the first two steps, as revealed by molecular dynamic analysis but is bent sharply by 70° in the lesion recognition complex^36^.

As introduced above earlier, the mammalian genomes contain the dinucleotide CpG clusters (CpG islands) in both promoter and exonic regions and these are very important regions for gene regulation abnormalities in diseases like cancer^39–43^. Methylation at cytosine C5 in the CpG islands represses gene expression and is an important epigenetic marker^44,45^, and inhibits the excision and repair of 8-oxoG mediated by OGG1^46^. The recognition and removal of 8-oxoG mechanism by a truncated version of human OGG1 has been very well studied by X-ray crystallography in the context of short DNA with a random sequence^34^. However, the operation of full-length human OGG1 on a CpG dinucleotide within a longer DNA sequence has not been investigated. Therefore, we set out to solve the cryo-EM structure of human OGG1 bound to a 35-bp DNA duplex with a C-8-oxoG at the center.

Guanine oxidation can occur at any position—in nucleosome-free regions as well as inside nucleosomes, yet existing structural studies on OGG1 have been based solely on naked DNA substrates^47^. A nucleosome core particle (NCP) is a pseudo two-fold symmetric structure composed of 147 bp of duplex wrapped 1.65 times around a histone octamer, which in turn is composed of two copies of histone H2A-H2B heterodimer and one copy of H3-H4 tetramer^48^. The 15 minor grooves of the nucleosome DNA are referred to as super-helical locations (SHLs), with the nucleosomal dyad axis site as SHL0, followed by SHL $\pm$1, $\pm$2, $\pm$3, $\pm$4, $\pm$5, $\pm$6 and $\pm$7, with the plus sites in the DNA entry side and minus sites at the DNA exit side^49–51^. In the absence of a chromatin remodeling complex, OGG1 is inactive toward 8-oxoG around the tightly packed SHL 0 site^52–54^. However, nucleosome DNA may transiently unwrap, particularly at the nucleosomal entry and exit sites. Indeed, OGG1 alone was partially active for 8-oxoG at the SHL-5 site^47^. Furthermore, we recently observed that OGG1 recruits NuRD (Nucleosome Remodeling and Deacetylase) complex component CHD4 to oxidative DNA damage sites both in vitro and in vivo^20^. Upon the introduction of DNA double-strand breaks, CHD4 recruits epigenetic modifiers, including DNA methyltransferases, to initiate de novo DNA methylation and gene silencing^20^. To understand how OGG1 recognizes the 8-oxoG in the context of an NCP, we introduced an 8-oxoG at the SHL-5 site and assembled a protein-DNA complex with a catalytically dead OGG1 (K249Q). We found by cryo-EM, interestingly, that OGG1 itself can bind meta-stably at multiple NCP sites, including the SHL-5, SHL+6, and the nucleosome entry and exit sites. We discuss throughout all the sections below, the potential biological implications of our observations.

## Results and discussion

### Cryo-EM structure of OGG1 bound to a 35-bp DNA containing the CpG dinucleotide

**Fig. 1:**
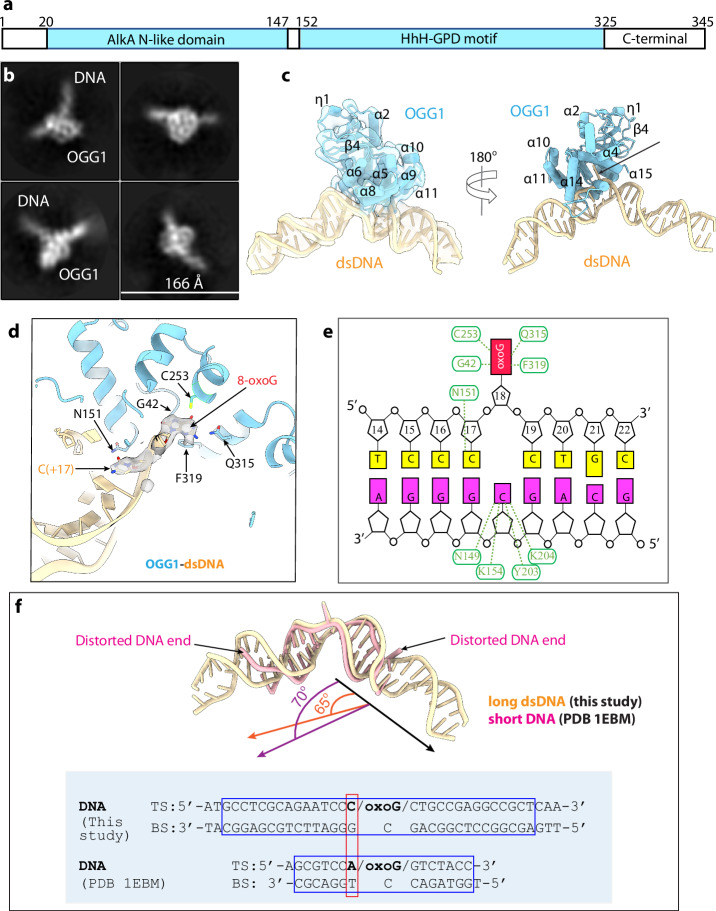
Cryo-EM structure of the OGG1 bound to a long naked DNA. **a** Domain architecture of the human OGG1. **b** Representative 2D class averages. **c** Cryo-EM structure shown in two side views, with the EM map superimposed and shown as transparent surface in the left panel. **d** The catalytic site of cryo-EM structure using a 35-bp DNA duplex reveals a flipped 8-oxoG. The interaction between the OGG1 N151 and the DNA C (+17) base, and the flipped 8-oxoG base are highlighted. **e** A schematic drawing of OGG1–DNA interaction. **f** Comparison of DNA bending in the OGG1–DNA EM structure and previous crystal structure. Inserted below is a comparison of the top sequence (TS) and bottom sequence (BS) of the DNA used in the current EM study and the previous crystal structure (PDB ID 1EBM).

Human OGG1 contains an AlkA_N-like domain and a helix-hairpin-helix (HhH-GPD) motif^55^ (Fig. 1a). Previous hOGG1-DNA structural studies used a relatively short (15-bp) DNA fragment and a truncated protein (aa 12–327) that removed 11 residues from the N-terminus and 18 residues from the C-terminus^34,56,57^. Of note, deletion of the N-terminal 11 residues abolished OGG1 transport to the mitochondria^58^. One motivation of the current study was to use a full-length hOGG1 with a longer DNA substrate because physiological DNA is virtually of infinite length. Furthermore, the CpG dinucleotide is concentrated in the CpG islands where, as we have introduced in diseases like cancer, epigenetic changes are important particularly in gene promoter and exonic regions of the genome^20,39–41,59,60^. Thus, we formed our rationale for studying the interaction between OGG1 with a longer DNA (35 bp, see “Methods”) with an unmethylated CpG dinucleotide, a natural substrate of de nova DNMTs. We used the K249Q mutant OGG1, which was previously shown by biochemical and X-ray crystal structural studies to lack catalytic activity but retain the ability to specifically recognize the 8-oxoG nucleotide^34,61^. We overexpressed and purified in *E. coli* a catalytically dead human OGG1(K249Q). We introduced an 8-oxoG at the center (-C16-p-8-oxoG17-) of a 35-bp GC-rich DNA duplex and mixed the purified protein and this DNA fragment at a molar ratio of 1.5:1 to assemble the OGG1–DNA complex. We then performed single-particle cryo-EM on the in vitro reconstituted complex. We obtained a cryo-EM 3D map of the complex at 3.6 Å average resolution (Fig. 1b, c, Supplementary Figs. 1, 2). By taking advantage of the crystal structure model of OGG1-DNA complex^34^, we solved the OGG1-DNA cryo-EM structure. The EM map had densities for atomic modeling for aa 12–325 of the 345-residue human protein and 33 bp of the 35-bp DNA. Therefore, the final atomic model misses 11 and 20 residues at the N- and C-terminus, respectively.

### OGG1 flips out the 8-oxoG and bends the dsDNA

Our cryo-EM structure shows that OGG1 binds tightly to the DNA minor groove and interacts with the sugar-phosphate backbone of the 8-oxoG-containing strand. The 8-oxoG base is fully flipped from the DNA helix and inserted deeply into the enzyme active pocket (Fig. 1d, e). This model of interaction and 8-oxoG flipping are similar to the previously reported crystal structures^34^. However, the DNA duplex is bent by 65°, 5° less bending than the previous crystal structure^34^ (Fig. 1f). The reduced bending in our structure is likely due to the enhanced local mechanical rigidity of the C-p-8-oxoG compared to the previously used A-p-8-oxoG, but our use of the two times longer DNA may have also contributed.

In our structure, the top DNA strand G42, and OGG1 residues Cys-253, Gln-315 and Phe-319 interact with 8-oxoG, and OGG1 residues Asn-149, Lys-154, Tyr-203, and Lys-204 interact with the “estranged” base cytosine in the complementary bottom DNA (Fig. 1d, e). These interactions are similar to the previous report structures with a shorter DNA^34^. Because our DNA substrate has a cytosine before 8-oxoG (the CpG dinucleotide), Asn-151 and C (+17) form a base-specific contact, equivalent to the Asn-151 and Adenosine interaction in the previous crystal structure^34^. The fact that OGG1 interacts well with both C-p-8-oxoG observed here and A-p-8-oxoG observed previously indicates that OGG1 can mold its catalytic pocket to interact with a different base immediately preceding the 8-oxoG. Such plasticity is expected of the enzyme.

### The positively charged OGG1 C-terminus contributes to DNA binding

**Fig. 2:**
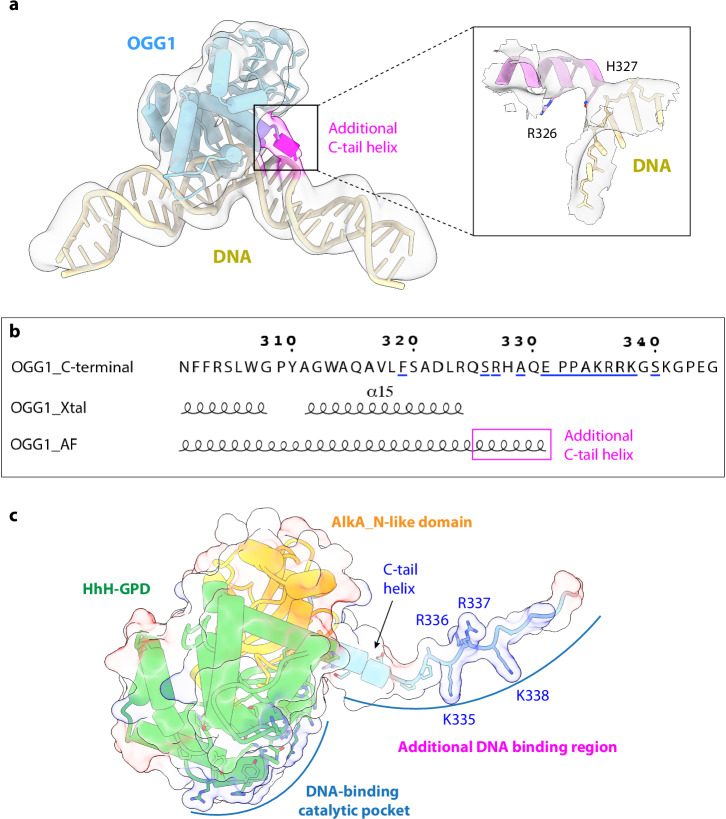
OGG1 C-terminal extension interacts with DNA. **a** The EM density of the OGG1 C-tail helix (magenta) adjacent to the DNA backbone. The 3D EM map is surface rendered at a very low threshold to visualize the weak C-tail helix density. The low threshold rendering makes the EM map appear at a lower resolution than the actual resolution of 3.6 Å. **b** The AphaFold2 prediction for the C-terminal of OGG1 shows a longer α-helix than the previous crystal structure assigned, which is consistent with the C-terminal extension density in OGG1–DNA cryo-EM 3D map. **c** Predicted additional DNA interaction region in OGG1.

Interestingly, we found in our EM map that the OGG1 C-terminal α-helix (the α15 helix, Fig. 1c) is seven residues (two helical turns) longer than the reported crystal structure^34^ (Fig. 2a, b). This extended helix is observed likely due to our use of the full-length protein and the longer DNA substrate. Although the local resolution is insufficient for atomic modeling, we found that AlphaFold2 also predicts a C-terminal α-helix of OGG1 one-turn longer than in the crystal structure^62^, and the predicted region includes the positively charged residues Arg-327 and His-328 that may interact with the negatively charged DNA phosphate backbone (Fig. 2b). Furthermore, the disordered C-terminus following the last predicted α-helix contains several additional positively charged residues (334-KRRKGSK-340); they likely also contribute to DNA binding. Indeed, A DNA binding site prediction by GraphSite showed that the positively charged C-terminus is conserved and is involved in DNA interaction^63^ (Fig. 2c).

**Fig. 3:**
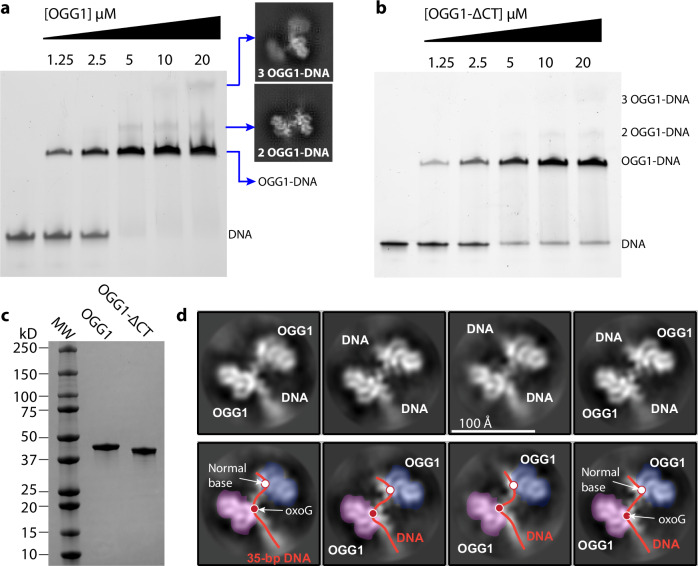
More than one OGG1 can bind to the single 8-oxoG-containing DNA, and the binding ability is reduced in the C-terminus truncated OGG1. **a** Formation of the OGG1-DNA complex was monitored by EMSA. 2.5 μM of 35-mer DNA duplex containing 8-oxoG at the middle base was mixed with OGG1 at the indicated concentrations. **b** EMSA was carried out with 8-oxoG DNA duplex using C-terminus deleted OGG1 (OGG1-$\triangle$CT) at the indicated concentrations. **c** SDS-PAGE gel of purified OGG1 and OGG1-$\triangle$CT used in the EMSA assay. **d** Four selected 2D class averages of two OGG1 molecules bound to the same linear DNA. The 35-bp dsDNA is bent twice, at the middle 8-oxoG (red dot) and near the end with a normal DNA base (white dot).

To assess the contribution of the OGG1 C-terminus to the DNA binding activity, we produced a C-terminal deleted OGG1 protein (OGG1-ΔCT), which removes all residues following Ser-326, i.e., removing the positively charged residues H328, K335, R336, R337, and K338. We performed electrophoretic mobility shift assay (EMSA) to compare the DNA binding of the wild type with the OGG1-ΔCT proteins. We found that the full-length OGG1 bound and shifted all free DNA up at the protein concentration of 5 μM or above, whereas the OGG1-ΔCT shifted only a portion of the DNA substrate even at the elevated protein concentration of 20 μM (Fig. 3a–c). The observed requirement of 5 μM OGG1 agrees with the recently reported apparent K_D_ of 2.22 μM on an undamaged DNA^64^. This result confirms that the strongly positively charged C-terminus contributes to OGG1’s DNA binding. Our observation is consistent with the previous observations that deletion of the OGG1 C-terminus reduces the enzyme’s affinity for DNA^34,65^.

Unexpectedly, we observed two super-shift bands in the EMSA gel with the full-length OGG1, and the corresponding bands are diminished with the OGG1-ΔCT (Fig. 3a–c). This observation indicates that two or even three copies of OGG1 can bind to the same 35-mer DNA simultaneously. Because there is only one 8-oxoG site in the middle of the 35-bp DNA, the one or two extra OGG1 must bind meta-stably to DNA regions that do not contain oxidative damage.

### OGG1 binds the dsDNA ends in a linear DNA substrate

To investigate where OGG1 binds beyond the middle 8-oxoG site in the linear DNA as revealed by the EMSA, we mixed OGG1 with the 35-bp DNA at a molar ratio of 10:1 and examined the assembled complexes by cryo-EM. We observed in many 2D class averages that two OGG1 molecules were able to bind to a single DNA substrate (Fig. 3d), and occasionally, three OGG1 were observed bound to a single DNA (Fig. 3a, top right insert). Careful inspection of the averaged images of the 2xOGG1-DNA complex reveals that one OGG1 is at the middle DNA damage site, and the other at one of the two DNA ends. Furthermore, the DNA appears to contain two kinks—a middle kink that must be from OGG1 flipping the middle 8-oxoG and bending DNA there, and a second kink at where the second OGG1 binds near the DNA end (Fig. 3d). Because only a single 8-oxoG was introduced to the center (+17) of the 35-bp dsDNA substrate, this apparent kink at the DNA end must be at an undamaged DNA site. Unlike the high-affinity tight binding at the 8-oxoG site, the OGG1 binding at the DNA end is weaker and more transient, with the possibility that the enzyme may be still moving on the DNA. Perhaps for this reason, we were unable to derive a reliable EM map of OGG1 at the DNA end.

### 8-oxoG does not appreciably distort the local DNA structure in a nucleosome

**Fig. 4:**
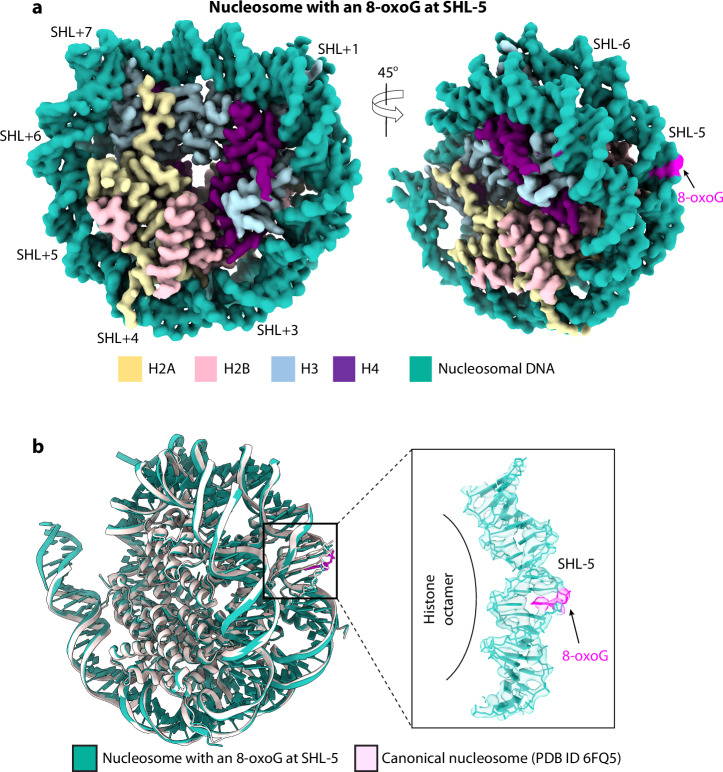
Structure of the nucleosome containing an 8-oxoG at the SHL-5 site. **a** Cryo-EM 3D map. **b** Superposition of the nucleosome with 8-oxoG and a canonical nucleosome (cyan, PDB ID 6FQ5). The right panel is a close-up view of the boxed 8-oxoG region around the SHL-5, superimposed with the EM density in transparent gray surface, showing that DNA structure at the 8-oxoG is not distorted and is virtually identical to the canonical nucleosomal DNA.

It is known that 8-oxoG does not distort the naked DNA duplex structure^66^. OGG1 has been shown to perform rapid one-dimensional searching for a damaged base through the genomic DNA without flipping each DNA base^67^. However, this work was done with naked DNA substrates, and it’s been unclear how OGG1 searches through nucleosomal DNA, and if the enzyme has any preferred binding site(s) on nucleosomes. Interestingly, OGG1 was recently found to have activity against nucleosomal substrates when the 8-oxoG lesions are located off the dyad axis, particularly at SHL-5 positioned opposite of the histones and out toward solvent^47^. This observation led us to wonder if an 8-oxoG appreciably distorts dsDNA in a nucleosome, given that the DNA has already been sufficiently bent by wrapping around the histone octamer. We inserted an 8-oxoG at the SHL-5 site of the 167-bp Widom 601 histone binding DNA sequence, reconstituted the nucleosome in vitro in the absence of the OGG1, and performed structural analysis of the 8-oxoG-containing nucleosome. We collected a cryo-EM dataset of 4414 micrographs and derived a 3.3-Å resolution EM map of the 8-oxoG-containing nucleosome (Fig. 4a, Supplementary Fig. 3). This resolution was sufficient to build an atomic model in the 3D map by referencing the well-established nucleosome structure^68^. We found that the local DNA structure at the 8-oxoG-containing SHL-5 site is virtually indistinguishable from the canonical nucleosome structure (Fig. 4a, b). Therefore, we conclude that 8-oxoG does not appreciably distort the local DNA structure even though the nucleosomal DNA is bent.

### The OGG1 occupancy at the nucleosomal DNA entry/exit sites is higher than that at the SHL-5 and SHL+6 sites

**Fig. 5:**
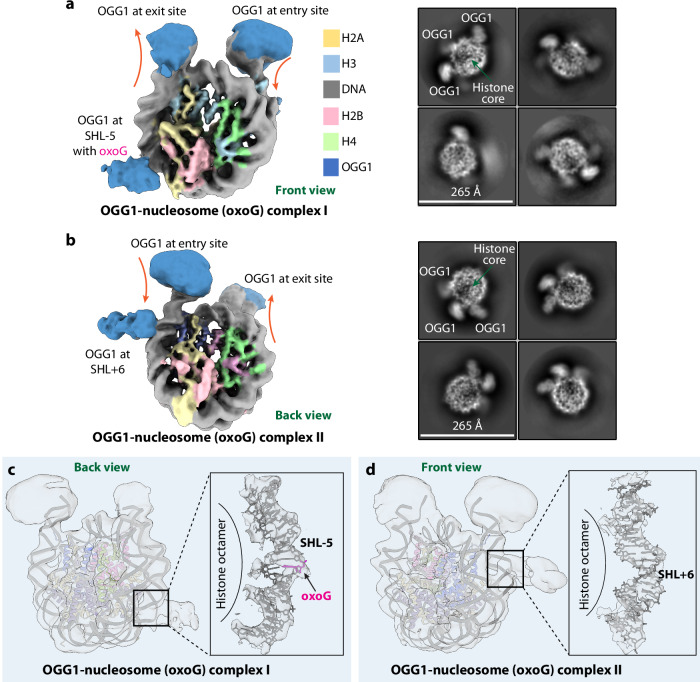
Cryo-EM structures of the OGG1-nucleosome (8-oxoG) in two distinct complexes. **a**, **b** Cryo-EM 3D maps of the OGG1–nucleosome in complex I (**a**) and complex II (**b**). The right panels show representative 2D averages of these two complexes. **c**, **d** Rigid-body fitting of the nucleosome (8-oxoG) structure into the 3D maps of complex I (**c**) and complex II (**d**). The right panels show the respective close-up views around the SHL DNA density where OGG1 binds. The nucleosomal DNA appears unaltered by OGG1 binding at either SHL-5 or SHL+6 sites.

To investigate if OGG1 interacts with nucleosomal DNA containing the 8-oxoG site, we used the above-described in vitro reconstituted nucleosome with an 8-oxoG lesion at the SHL-5 site and launched an extensive effort to assemble the OGG1–nucleosome complex for structural analysis. We found by cryo-EM imaging that simply mixing OGG1 and nucleosome (0.9 mg/mL) at various molar ratios ranging from 5:1 to 10:1 did not lead to a stable complex and that the addition of the commonly used crosslinking agent glutaraldehyde at 0.1% concentration did not help the assembly either. Finally, we used 0.2% formaldehyde which is widely used for protein-DNA crosslinking and found by cryo-EM that about 1.5% of the nucleosome particles formed complexes with OGG1 at the sites of SHL-5 (0.4%) and SHL+6 (1.1%) (Supplementary Fig. 4). 2D and 3D classifications in RELION^69^ indicated that OGG1 binds preferentially at the entry/exit site DNA (3.0%) but less frequently at internal DNA sites (1.5%). Further analysis by CryoDRGN^70^ on a subset of the OGG1–nucleosome complex particles derived from 3D classification led to two new 3D EM maps, complexes I and II, at 5.7 and 7.6 Å overall resolutions, respectively (Fig. 5a, b, Supplementary Figs. 4–6).

In complex I, OGG1 binds at both entry and exit DNA sites as well as at the SHL-5 site, which contained the 8-oxoG lesion (Fig. 5a), and in complex II, OGG1 also binds at both entry and exit DNA sites, but surprisingly, at the SHL+6 site that did not contain the 8-oxoG lesion (Fig. 5b). In both complex maps, the EM densities for the bound OGG1 were of low resolution at the entry and exit sites, and the OGG1 only had partial densities at the SHL-5 and SHL+6 sites. At all binding sites observed, OGG1 has little contact with the histone core and only interacts with nucleosomal DNA. At the SHL-5 and SHL+6 sites, OGG1 engages the nucleosome at the DNA minor grooves. The observed OGG1 preference for the nucleosome entry/exit sites is likely a result of the enzyme getting stuck at these DNA ends. The DNA entry/exit sites of an in vitro reconstituted single nucleosome resemble double-strand DNA breaks (DSB). We suggest that OGG1’s preference for these sites may endow the enzyme to function by marking the DSB sites in vivo. OGG1 scans the genome at a speed approaching one-dimensional diffusion limit^67^ and is well positioned to rapidly zoom onto and protect the double-strand DNA ends as soon as a DSB event occurs. Indeed, one study found that OGG1 is targeted to the DSB sites induced by either ionizing radiation or gamma rays^71^ and another study reported that OGG1 protects cells from methylmercury-induced DSB damage^72^.

We next analyzed if OGG1 binding at the SHL sites alters the local nucleosomal DNA at these sites. Although OGG1 is of low resolution, the nucleosome is rigid and much better resolved, such that the DNA major and minor grooves are visualized, enabling an accurate docking with the above-described nucleosome (8-oxoG) structure determined in the absence of OGG1 (Fig. 5c, d). Based on the docking result, the nucleosomal DNA is not distorted, and the DNA base does not appear to have flipped at either SHL-5 or SHL+6 site, although the local resolution is insufficient to make a conclusion. This structural observation is consistent with the previous reports that the OGG1 activity is either completely inhibited or dramatically decreased in the context of a nucleosome^47,52^.

Our observed OGG1 binding at the nucleosome SHL+6 site (lacking 8-oxoG) may be equivalent to the previously observed interrogation complex entrapped on a linear DNA by intermolecular crosslinking, and the OGG1 binding at the nucleosomal SHL-5 (with 8-oxoG) may be equivalent to the encounter complex also entrapped on a linear DNA^36^. At these initial binding states, the local DNA structure is largely undisturbed and relatively straight. The OGG1 binding at these sites must be weak and transient because crosslinking had to be used in the previous study with the linear DNA^36^ as well as in the current study with a nucleosome.

The possibility that OGG1 binds but does not flip the 8-oxoG at the nucleosomal SHL-5 site suggests that OGG1 needs to partner with a chromatin remodeler to excise 8-oxoG in the nucleosomal region. Such chromatin remodelers may include RSC, SWI/SNF^53^, and NuRD^20^. Indeed, OGG1 physically interacts with CHD4, a key component of NuRD^20^. RSC was shown to be required for the excision of 8-oxoG located within nucleosomal DNA^54^. Furthermore, our finding that OGG1 can weakly bind—perhaps meta-stably—at the SHL sites may further suggest that chromatin remodelers do not need to move an 8-oxoG all the way out of the nucleosome, and instead may only need to scrunch and shift the 8-oxoG region to a nearby SHL site, where the damaged base can be removed by OGG1.

### OGG1 bends the entry site nucleosomal DNA and flips an undamaged G

**Fig. 6:**
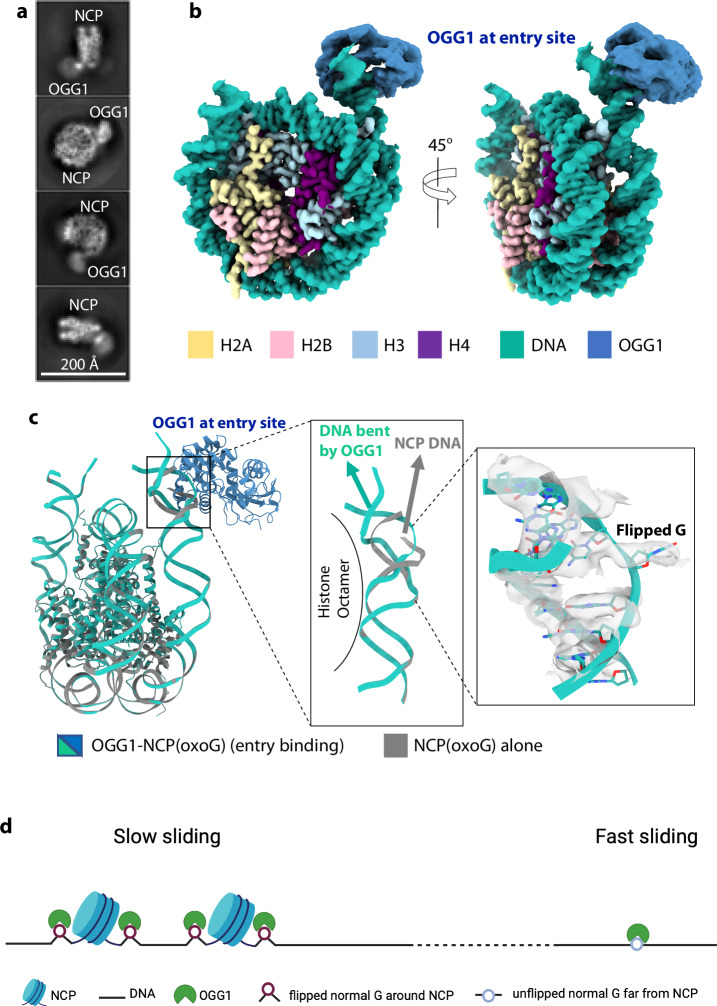
OGG1 binding at the nucleosome entry site bends DNA and flips a guanosine base. **a** Selected 2D class averages of nucleosome particles with clear OGG1 binding at the entry site. **b** Composite EM map of the OGG1–nucleosome complex. The histone core and DNA are resolved to 3.2 Å, but the OGG1 is at a lower resolution of 5–7 Å. **c** Atomic model of the nucleosome with OGG1 at the entry site in cartoons. The zoomed windows show that OGG1 bends the local DNA and flips the G near the end of the nucleosome DNA. **d** A proposed OGG1 interrogation mechanism. OGG1 slides rapidly in unobstructed linear DNA regions. In the fast-sliding mode, OGG1 searches for 8-oxoG without flipping the bases. In highly crowded regions where DNA is packed into nucleosomes and bound by additional chromatin proteins, OGG1 may move slowly. And in the slow sliding mode, OGG1 flips the bases to search for 8-oxoG.

We used a 167-bp DNA in our reconstituted nucleosome (“Methods”). Because only 147 bp DNA is tightly wrapped around the histone core, there is 10-bp free DNA at both entry and exit sites of the nucleosome. As shown above, OGG1 prefers to bind at these sites over the internal SHL-5 and +6 sites, therefore, we decided to perform a focused 3D classification in the OGG1-bound DNA entry region and derived an EM map at 3.2 Å average resolution (Fig. 6a, b, Supplementary Fig. 7). In this map, the nucleosome region including the DNA is resolved at high resolution, but the OGG1 is only at a low resolution of 5–7 Å. Despite the low resolution, OGG1 has full density in the composite EM map (Fig. 6b).

We found that the OGG1-bound DNA region at the nucleosomal entry site is bent, as compared with a free nucleosome structure in which DNA protrudes straightly outward (Fig. 6c, Supplementary Fig. 8). This is unexpected because the region contains no 8-oxoG, DNA is largely straight in the crystal structure of the interrogation and encounter complexes in which OGG1 is bound to an undamaged DNA^36^, and normal DNA bases are not expected to be flipped during rapid OGG1 sliding on DNA^67^. The sharply bent DNA was observed previously only in the recognition complex in the presence of the damaged base 8-oxoG in which the 8-oxoG had been flipped. Consistent with sharp DNA bending, we find the undamaged base G closest to the OGG1 catalytic site has been flipped out to an extrahelical region (Fig. 6c).

## Discussion

OGG1 is only 38 kDa and a little over 60 kDa when bound to a 35-bp dsDNA. Our 3.6-Å EM map of the OGG1–DNA complex demonstrates the utility of cryo-EM in analyzing low molecular weight DNA binding proteins. Our study has revealed that the human OGG1 binds and bends a linear and long DNA containing an oxidized CpG dinucleotide (C-p-8-oxoG) in a manner highly similar to the previously revealed binding on a short DNA and that the positively charged OGG1 C-terminus not only contributes to the DNA binding affinity but also induces DNA super-shifts, due to the binding at the dsDNA end(s) that do not contain a damage site. We have shown that an 8-oxoG lesion at SHL-5 does not appreciably distort the human nucleosomal DNA, and OGG1 prefers to bind to DNA ends at the entry and exit sites. The OGG1 binding at the end of the naked dsDNA is likely related to the OGG1 binding at the dsDNA ends at the nucleosomal DNA entry and exit sites. We suggest that such preference may be related to OGG1’s role in the DSB repair, a function implicated in previous cellular studies^71,72^. This study has further revealed two unexpected properties of the human OGG1: the enzyme bends the DNA and flips out an undamaged guanine base at the nucleosomal entry site and can bind meta-stably to internal nucleosomal DNA at the SHL sites such as the -5 and +6 sites.

We found that the nucleotide likely is not flipped out by the OGG1 at the internal nucleosomal SHL sites. The OGG1 binds only meta-stably and is likely wobbly at these sites, resulting in the low local resolution for the enzyme (Fig. 5a–d). Therefore, the nucleotide base position could not be determined in the OGG1’s catalytic pocket. However, the nucleosome and associated DNA were stable and had much better resolution, with well-resolved major and minor grooves. Because there was no density gap in the EM map of the nucleosomal DNA, we infer that DNA bases at the internal SHL sites are not flipped by OGG1. Although we used the inactive K249Q mutant OGG1 in this study, it is unlikely that the mutation was the reason for these internal nucleosomal DNA bases not being flipped out. Indeed, we found the mutant can flip the DNA base in the naked DNA as well as in the nucleosomal DNA entry and exit sites, in agreement with the previously reported base flipping activity of the mutant enzyme^34^. OGG1’s likely inability to flip DNA bases inside the nucleosome agrees with the previous observation that OGG1 has very limited access to 8-oxoG in the context of NCP^52^ and that the enzyme alone cannot effectively process the 8-oxoG in the NCP in vitro, and therefore, a nucleosomal remodeler is likely required^47,52–54^.

In a recent study, Zheng et al. showed by cryo-EM that the human alkyladenine DNA glycosylase (AAG) could access the DNA damage sites (deoxyinosine) in the nucleosome^73^. It is unclear if the mechanistic difference with OGG1 is enzyme-specific or due to the different types of damage that the two enzymes process. At a minimum, deoxyinosine itself causes a global nucleosomal DNA perturbation^73^, in contrast to 8-oxoG, which not only maintains normal G:C base pairing but also causes no appreciable DNA distortion (Fig. 4b).

Whether OGG1 slides on DNA without flipping bases or simultaneously flips the bases along the way is an important unresolved question. In a previous crystallographic study, an undamaged G was covalently trapped in an extruded/flipped mode without fully engaging the OGG1 catalytic pocket^74^. However, it has been unclear if OGG1 extrudes a normal G in the absence of a trapping crosslinker. Our above-described OGG1–nucleosome structure revealing a flipped normal G at the nucleosome entry site was determined in the absence of a specific crosslinker (Fig. 6c). However, the nonspecific protein-DNA crosslinker formaldehyde was used to stabilize the OGG1–nucleosome complexes. In the absence of any crosslinking agent, we found that OGG1 can bind the free dsDNA ends and sharply bend the DNA there (Fig. 3d). The DNA bending at the end of the naked DNA may suggest that the bases are flipped there. Taken together, we suggest that OGG1 may flip the normal DNA bases when its movement on DNA is hindered or slowed down, such as at the DNA ends.

However, a previous single molecule study revealed that DNA slides on DNA too rapidly to bend DNA and flip DNA bases^67^. To reconcile the single molecule result with the current cryo-EM observations that OGG1 bends and likely extrudes undamaged bases at the nucleosomal or naked DNA ends, we suggest that OGG1 sliding on DNA may be context dependent—the enzyme must be sliding rapidly on unobstructed linear DNA regions, but the enzyme slows down in crowded regions, such as in-between nucleosomes or near other DNA binding proteins, or at the DSBs. It is perhaps at these slowly sliding regions that OGG1 can bend DNA and flip the bases (Fig. 6d). Indeed, in our sample preparations, the OGG1 at the nucleosome entry site is somewhat trapped between the free dsDNA end and the nucleosome region, and OGG1 at the 35-bp DNA end is trapped between the dsDNA end and a stably bound OGG1 at the 17th 8-oxoG site.

Finally, we note that further investigation is required to fully address the OGG1 base flipping during DNA sliding and base interrogation, and how chromatin remodelers coordinate with OGG1 to process oxidized DNA bases in the nucleosomal internal sites.

## Methods

### Preparation of the human OGG1 and the DNA substrate

Expression and purification of the full-length hOGG1 carrying the K249Q mutation and an N-terminal 6His-tag was as described^34^. Briefly, the full-length sequence with the K249Q mutation was cloned into the pET28b plasmid and overexpressed in *E. coli* BL21. Cells were grown in LB medium supplemented with kanamycin (50 μg/mL). Protein expression was induced when the cell density reached the OD_600_ of ∼0.8 by adding 0.3 mM Isopropyl β-D-1-thiogalactopyranoside. Induced cells were grown overnight at 16 °C prior to harvesting by centrifugation. The cells were lysed by sonication in a solution of 50 mM Tris-HCl pH 7.5, 500 mM NaCl, 10 mM imidazole, and 5% glycerol (buffer A). The protein was immobilized by Ni-NTA resin (Qiagen) and eluted with a buffer of 20 mM MES (pH 6.0), 100 mM NaCl, 300 mM imidazole and 5% glycerol. The eluted fractions were applied to a Resource S column (GE Lifesciences) equilibrated with buffer B containing 20 mM MES (pH 6.0), 100 mM NaCl, and 5 mM β-mercaptoethanol (βΜΕ). The proteins were eluted with a salt gradient to 1 M NaCl in buffer B. Protein fractions pure in hOGG1 were identified by SDS-PAGE gel, pooled, and concentrated using a 10 kDa cutoff centrifugal filter (Amicon, Millipore). Protein was further purified by Superdex-75 gel filtration chromatography (GE Healthcare) equilibrated with 20 mM HEPES pH 7.4, 300 mM NaCl, 6 mM βME, and 5% glycerol and stored at −80 °C. Synthetic oligonucleotides containing an 8-oxoG base at the center of 35 nucleotides (5′-ATGCCTCGCAGAATCCC/i8oxodG/CTGCCGAGGCCGCTCAA and 5′-TACGGAGCGTCTTAGGGCGACGGCTCCGGCGAGTT) were purchased from Integrated DNA Technologies (IDT). The duplex DNA was assembled by thermal annealing. To assemble the OGG1-DNA complex, purified OGG1 protein and annealed dsDNA were mixed in 1.5:1 or 5:1 (protein over DNA) molar ratios in 20 mM HEPES buffer at pH 7.4, 100 mM NaCl, 5 mM βΜΕ on ice.

### Electrophoretic mobility shift assay

DNA binding with full-length OGG1 and C-terminus truncated OGG1-$\triangle$C (truncated from Ser326 to the last amino acid Gly345) was monitored by electrophoretic mobility shift assay (EMSA). For EMSA experiments, OGG1 or OGG1-$\triangle$C was mixed with 2.5 μM of an 8-oxoG modified DNA duplex (5′-ATGCCTCGCAGAATCCC/i8oxodG/CTGCCGAGGCCGCTCAA and 5′-TACGGAGCGTCTTAGGGCGACGGCTCCGGCGAGTT) at the indicated concentrations for 30 min on ice in binding Buffer (40 mM HEPES pH 7.4, 50 mM NaCl, 5 mM βME, and 5% glycerol). OGG1–DNA complexes were separated from free DNA on a 4–20% polyacrylamide native gel purchased from BioRad, and the individual bands were visualized on a ChemiDoc MP imaging system and analyzed with Image Lab software.

### Nucleosome preparation and assembly of the OGG1–nucleosome complex

The vector pET29a-YS14 containing the four *Xenopus laevis* histones was a gift from Jung-Hyun Min (Addgene plasmid #66890; http://n2t.net/addgene:66890; RRID: Addgene_66890). *Xenopus laevis* histones and the 167-bp DNA fragments for nucleosome reconstitution were prepared as described^75–77^. Briefly, the vector containing the Widom 601 sequence was a gift from Dr. Tinghai Xu (Van Andel Institute). The vector was used as a template for large-scale (25 mL) PCR reactions with two primers synthesized by IDT (forward primer: ATCGGCCGCCCTGGAGAATCCCGGTGCCGAGGCC/8-oxoG/CTCAATTGGTC, reverse primer: ATCGGCCGCCACAGGATGTATATATCTGAC). The PCR products were purified by Resource Q 6 ml (GE Healthcare) ion exchange chromatography^78^.

Nucleosome reconstitution was performed as described^75^. Briefly, purified histone octamer and DNA were mixed at 1:1 molar ratio in 2 M KCl and transferred to Slide-A-Lyzer MINI Dialysis Units (20,000 MWCO, Thermo Scientific). The sample was gradient dialyzed against low salt buffer (50 mM KCl, 20 mM HEPES pH 7.5, 1 mM EDTA pH 8.0, 5 mM βΜΕ) over 18 h. The sample was moved into a low salt buffer (50 mM KCl, 20 mM HEPES pH 7.5, 1 mM EDTA pH 8.0, 5 mM βΜΕ) and dialyzed for another hour, and finally stored at 4 °C. The concentration of the reconstituted nucleosome was monitored by measuring absorbance at 254 nm by Nanodrop (Thermo Scientific).

To prepare the OGG1–nucleosome complex, purified OGG1 and reconstituted nucleosome were mixed at a molar ratio of 5:1 and dialyzed against buffer (50 mM NaCl, 20 mM HEPES pH 7.5, pH 8.0, 5 mM βΜΕ). The dialyzed mixture containing nucleosome core particle and OGG1 were crosslinked with 0.2% (v/v) formaldehyde for 10 min on ice. The crosslinking reaction was quenched by 40 mM Tris-HCl pH 7.5 (final concentration) for 10 min. The sample was transferred to a Slide-A-Lyzer MINI Dialysis Unit (20,000 MWCO, Thermo Scientific) and dialyzed for 2 h against a 600-mL dialysis buffer (50 mM NaCl, 20 mM HEPES pH 7.4, 5 mM βΜΕ). The sample was subsequently concentrated to a final concentration of around 0.9 mg/mL.

### Cryo-electron microscopy

For cryo-EM grids preparation, 2.5-μL aliquots of pre-assembled hOGG1-DNA complex or hOGG1–nucleosome at a concentration of about 0.2 mg/mL were placed on glow-discharged holey carbon grids (Quantifoil Au R2/2, 300 mesh) and were flash-frozen in liquid ethane using a Vitrobot Mark IV (FEI). Cryo-EM data was collected automatically with SerialEM in a 300-kV FEI Titan Krios electron microscope with defocus values from −1.0 to −2.0 μm^79^. The microscope was operated with a K3 direct detector at a nominal magnification of 130,000× and a pixel size of 0.414 Å per pixel. The dose rate was 8 electrons per Å^2^ per second, and the total exposure time was 8 s.

### Cryo-EM image processing

Program MotionCorr 2.1 was used for motion correction of the raw movie micrographs, and CTFFIND 4.1 was used for estimating and correcting contrast transfer function in each micrograph^80,81^. All remaining steps were performed using RELION-3.1, cryoSPARC and CryoDRGN^69,70,82^. The resolution of the map was estimated by the gold-standard Fourier shell correlation at a correlation cutoff value of 0.143.

For the OGG1–DNA complex with an 8-oxoG at the middle of the 35-bp DNA substrate, we collected 11,614 raw movie micrographs, and split the whole micrographs into 12 parts (Supplementary Fig. 1). Around 1.5 million particles were picked automatically from each part. After 2D classification in cryoSPARC, around 1 million particles from each part were selected and used for ab initio 3D reconstruction in cryoSPARC. Based on the quality of the second round of starting 3D maps, a final dataset of 787,683 particles was selected and combined for further non-uniform refinement in CryoSPARC, resulting in a 3.6-Å average resolution 3D map (Supplementary Figs. 1, 2).

For the nucleosome containing an 8-oxoG at the SHL-5 site, we collected 4414 raw movie micrographs. A total of 2,699,710 particles were picked automatically. After 2D classification, a total of 1,694,674 particles were selected and used for 3D classification. Based on the quality of the four 3D classes, 430,285 particles were retained for further 3D reconstruction, refinement, and postprocessing, resulting in a 3D map at an overall resolution of 3.3 Å (Supplementary Fig. 3).

For the OGG1–nucleosome complex with an 8-oxoG at the SHL-5 site, we collected 17,124 raw movie micrographs. A total of 14,501,354 particles were picked automatically. After 2D classification, a total of 2,263,164 particles were selected and used for 3D classification. Based on the quality of the four 3D classes, 1,578,612 particles showing good NCP shape were retained for further 3D reconstruction and refinement (Supplementary Fig. 4). We applied cryoDRGN (version 0.3.2) to distinguish different states of complex following the default protocols. Particles were extracted and down-sampled to 64 × 64 pixels for cryoDRGN analysis. The results were visualized by the Uniform Manifold Approximation and Projection (UMAP) method (Supplementary Fig. 5a) and clustered into 20 groups to represent the heterogeneity. The particles were categorized into two major states (Supplementary Fig. 5a). One group contained particles of the nucleosome alone, while the other showed clear binding protein density (Supplementary Fig. 5b). The OGG1 were found to attach predominantly at the DNA entry sites (Supplementary Fig. 5b). Two groups show additional OGG1 binding sites on nucleosome (group 3 and 4, Supplementary Fig. 5b). The unbinned particles were then selected for 3D refinement in Relion, resulting in a 5.7-Å average resolution 3D map with OGG1 bound at the entry/exit and the SHL+5 sites (Supplementary Figs. 4–6) and a 7.6-Å average resolution 3D map with OGG1 bound at the entry/exit and SHL+6 sites (Supplementary Figs. 4–6).

We further performed a focused refinement on the OGG1 bound to the entry site DNA on the same OGG1–nucleosome dataset described above (Supplementary Fig. 7). We combined the particles showing good shape of NCP and OGG1 binding. Then we applied a mask around OGG1 and performed a focused 3D classification without alignment in RELION and selected three classes of 439,090 particles with clear OGG1 occupancy at the nucleosome entry site for a subsequent 3D auto refinement. We finally obtained a 3D map at an overall resolution of 3.2 Å for the nucleosome with OGG1 bound at the entry site.

### Structural modeling, refinement, and validation

We used the published crystal structure of human OGG1-DNA (PDB ID 1EBM) and the cryo-EM structure of the *Xenopus laevis* nucleosome (PDB ID 6FQ5) as initial models. These models were docked into their respective EM maps and were manually corrected or rebuilt for local fitting with the densities in COOT and Chimera^83,84^. The complete models of the human OGG1–DNA, the nucleosome, and the OGG1–nucleosome complexes were refined by real-space refinement in the PHENIX program and subsequently adjusted manually in COOT. Finally, all models were validated using MolProbity in PHENIX^85^. We used information derived from a high-resolution structure to resolve the −/+ SHLs (i.e., the 180 ambiguity) in the low-resolution maps of complexes I and II. We first assigned the SHLs in the 3.2-Å structure of OGG1 bound at the nucleosome entry site (Fig. 6). Determination of this structure indicates that OGG1 prefers the entry site over the exit site. In the low-resolution OGG1-nucleosome complex I and II structures (Fig. 5), we resolved the 180° uncertainty and assigned the SHL-5 location based on the knowledge that the end with a stronger OGG1 density is the entry site, and the end with a weaker OGG1 density is the exit site. DNA bending angle in the OGG1-DNA complex was measured using DNA-bending-angle script in GitHub (https://github.com/Sunyp-IM/DNA-bending-angle). Structural figures were prepared in ChimeraX^86^. The schematic diagram for OGG1 interrogation mechanism was created in BioRender (You, Q. (2024) BioRender.com/r12h908).

### Reporting summary

Further information on research design is available in the Nature Portfolio Reporting Summary linked to this article.

## Supplementary information

Supplementary information

Reporting summary

## Data Availability

The EM 3D map of the OGG1-DNA (8-oxoG) complex at 3.6 Å average resolution and the associated PDB coordinates have been deposited in the RCSB Protein Data Bank with accession codes EMD-43607 and PDB ID 8VX4, respectively. The EM map of the nucleosome containing an 8-oxoG at SHL-5 site at 3.3 Å average resolution and associated PDB coordinates have been deposited in the RCSB Protein Data Bank with accession codes EMD-43608 and PDB ID 8VX5, respectively. The EM map of OGG1 bound to the nucleosome entry site at 3.2 Å average resolution has been deposited in the RCSB Protein Data Bank with accession codes EMD-43609 and PDB ID 8VX6. The EM maps of OGG1 bound at the entry/exit and SHL-5 sites at 5.7 Å average resolution and of OGG1 bound at the entry/exit and the SHL+6 sites at 7.6 Å average resolution have been deposited in the RCSB Protein Data Bank with accession codes EMDB-43610 and EMDB-43611, respectively. Uncropped and unedited gel images are shown in Supplementary Fig. 9a–c.

## Materials availability

All plasmids for expressing the OGG1 proteins in *E. coli* used in this study are available by contacting the corresponding authors.

## References

[1] Friedberg, E. C., Walker, G. C., Siede, W. & Wood, R. D. *DNA Repair and Mutagenesis* (American Society for Microbiology Press, 2005).

[2] Chatterjee, N. & Walker, G. C. Mechanisms of DNA damage, repair, and mutagenesis. *Environ. Mol. Mutagen.* **58**, 235–263 (2017). 28485537 10.1002/em.22087 PMC5474181

[3] Friedberg, E. C. DNA damage and repair. *Nature* **421**, 436–440 (2003). 12540918 10.1038/nature01408

[4] Radak, Z. & Boldogh, I. 8-Oxo-7,8-dihydroguanine: links to gene expression, aging, and defense against oxidative stress. *Free Radic. Biol. Med.* **49**, 587–596 (2010). 20483371 10.1016/j.freeradbiomed.2010.05.008 PMC2943936

[5] Burrows, C. J. Oxidative nucleobase modifications leading to strand scission. *Chem. Rev.* **98**, 1109–1151 (1998). 11848927 10.1021/cr960421s

[6] Candeias, L. P. & Steenken, S. Reaction of HO* with guanine derivatives in aqueous solution: formation of two different redox-active OH-adduct radicals and their unimolecular transformation reactions. Properties of G(-H)*. *Chemistry* **6**, 475–484 (2000). 10747414 10.1002/(sici)1521-3765(20000204)6:3<475::aid-chem475>3.0.co;2-e

[7] Ba, X. et al. The role of 8-oxoguanine DNA glycosylase-1 in inflammation. *Int. J. Mol. Sci.* **15**, 16975–16997 (2014). 25250913 10.3390/ijms150916975 PMC4200771

[8] Dizdaroglu, M. Formation of 8-hydroxyguanine moiety in deoxyribonucleic acid on γ-irradiation in aqueous solution. *Biochemistry* **24**, 4476–4481 (1985). 4052410 10.1021/bi00337a032

[9] Grollman, A. P. & Moriya, M. Mutagenesis by 8-oxoguanine: an enemy within. *Trends Genet.* **9**, 246–249 (1993). 8379000 10.1016/0168-9525(93)90089-z

[10] Akiyama, M., Maki, H., Sekiguchi, M. & Horiuchi, T. A specific role of MutT protein: to prevent dG.dA mispairing in DNA replication. *Proc. Natl Acad. Sci. USA* **86**, 3949–3952 (1989). 2657730 10.1073/pnas.86.11.3949 PMC287365

[11] Shibutani, S., Takeshita, M. & Grollman, A. P. Insertion of specific bases during DNA synthesis past the oxidation-damaged base 8-oxodG. *Nature* **349**, 431–434 (1991). 1992344 10.1038/349431a0

[12] Michaels, M. L., Pham, L., Cruz, C. & Miller, J. H. MutM, a protein that prevents G C→T A transversions, is formamidopyrimidine-DNA glycosylase. *Nucleic Acids Res.* **19**, 3629–3632 (1991). 1649454 10.1093/nar/19.13.3629 PMC328390

[13] David, S. S., O’Shea, V. L. & Kundu, S. Base-excision repair of oxidative DNA damage. *Nature* **447**, 941–950 (2007). 17581577 10.1038/nature05978 PMC2896554

[14] Krokan, H. E. & Bjørås, M. Base excision repair. *Cold Spring Harb. Perspect. Biol.* **5**, a012583 (2013). 10.1101/cshperspect.a012583 PMC3683898 23545420

[15] Izumi, T. et al. Mammalian DNA base excision repair proteins: their interactions and role in repair of oxidative DNA damage. *Toxicology* **193**, 43–65 (2003). 14599767 10.1016/s0300-483x(03)00289-0

[16] Dizdaroglu, M., Kirkali, G. & Jaruga, P. Formamidopyrimidines in DNA: mechanisms of formation, repair, and biological effects. *Free Radic. Biol. Med.* **45**, 1610–1621 (2008). 18692130 10.1016/j.freeradbiomed.2008.07.004

[17] Hart, R. W. & Setlow, R. B. Correlation between deoxyribonucleic acid excision-repair and life-span in a number of mammalian species. *Proc. Natl Acad. Sci. USA* **71**, 2169–2173 (1974). 4526202 10.1073/pnas.71.6.2169 PMC388412

[18] Ames, B. N., Shigenaga, M. K. & Hagen, T. M. Oxidants, antioxidants, and the degenerative diseases of aging. *Proc. Natl Acad. Sci. USA* **90**, 7915–7922 (1993). 8367443 10.1073/pnas.90.17.7915 PMC47258

[19] Ba, X., Aguilera-Aguirre, L., Sur, S. & Boldogh, I. 8-Oxoguanine DNA glycosylase-1-driven DNA base excision repair: role in asthma pathogenesis. *Curr. Opin. Allergy Clin. Immunol.* **15**, 89–97 (2015). 25486379 10.1097/ACI.0000000000000135 PMC4364697

[20] Xia, L. et al. CHD4 has oncogenic functions in initiating and maintaining epigenetic suppression of multiple tumor suppressor genes. *Cancer Cell* **31**, 653–668.e7 (2017). 28486105 10.1016/j.ccell.2017.04.005 PMC5587180

[21] Donley, N. et al. Small molecule inhibitors of 8-oxoguanine DNA glycosylase-1 (OGG1). *ACS Chem. Biol.* **10**, 2334–2343 (2015). 26218629 10.1021/acschembio.5b00452 PMC4894821

[22] Tahara, Y.-K. et al. Potent and selective inhibitors of 8-oxoguanine DNA glycosylase. *J. Am. Chem. Soc.* **140**, 2105–2114 (2018). 29376367 10.1021/jacs.7b09316 PMC5823510

[23] Visnes, T. et al. Small-molecule inhibitor of OGG1 suppresses proinflammatory gene expression and inflammation. *Science* **362**, 834–839 (2018). 30442810 10.1126/science.aar8048 PMC6645780

[24] Baptiste, B. A. et al. Enhanced mitochondrial DNA repair of the common disease-associated variant, Ser326Cys, of hOGG1 through small molecule intervention. *Free Radic. Biol. Med.* **124**, 149–162 (2018). 29879444 10.1016/j.freeradbiomed.2018.05.094 PMC6098717

[25] Schniertshauer, D. et al. The activity of the DNA repair enzyme hOGG1 can be directly modulated by ubiquinol. *DNA Repair* **87**, 102784 (2020). 31923624 10.1016/j.dnarep.2019.102784

[26] Oka, S. et al. MTH1 and OGG1 maintain a low level of 8-oxoguanine in Alzheimer’s brain, and prevent the progression of Alzheimer’s pathogenesis. *Sci. Rep.* **11**, 5819 (2021). 33758207 10.1038/s41598-021-84640-9 PMC7988129

[27] Komakula, S. S. B. et al. The DNA repair protein OGG1 protects against obesity by altering mitochondrial energetics in white adipose tissue. *Sci. Rep.* **8**, 14886 (2018). 30291284 10.1038/s41598-018-33151-1 PMC6173743

[28] Komakula, S. S. B., Blaze, B., Ye, H., Dobrzyn, A. & Sampath, H. A novel role for the DNA repair enzyme 8-oxoguanine DNA glycosylase in adipogenesis. *Int. J. Mol. Sci.* **22**, 1152 (2021). 10.3390/ijms22031152 PMC7865743 33503804

[29] Michel, M. et al. Small-molecule activation of OGG1 increases oxidative DNA damage repair by gaining a new function. *Science* **376**, 1471–1476 (2022). 35737787 10.1126/science.abf8980

[30] Nishioka, K. et al. Expression and differential intracellular localization of two major forms of human 8-oxoguanine DNA glycosylase encoded by alternatively spliced OGG1 mRNAs. *Mol. Biol. Cell* **10**, 1637–1652 (1999). 10233168 10.1091/mbc.10.5.1637 PMC30487

[31] Takao, M., Aburatani, H., Kobayashi, K. & Yasui, A. Mitochondrial targeting of human DNA glycosylases for repair of oxidative DNA damage. *Nucleic Acids Res.* **26**, 2917–2922 (1998). 9611236 10.1093/nar/26.12.2917 PMC147628

[32] Ba, X. & Boldogh, I. 8-Oxoguanine DNA glycosylase 1: beyond repair of the oxidatively modified base lesions. *Redox Biol.* **14**, 669–678 (2018). 29175754 10.1016/j.redox.2017.11.008 PMC5975208

[33] Hazra, T. K. et al. Oxidative DNA damage repair in mammalian cells: a new perspective. *DNA Repair* **6**, 470–480 (2007). 17116430 10.1016/j.dnarep.2006.10.011 PMC2702509

[34] Bruner, S. D., Norman, D. P. & Verdine, G. L. Structural basis for recognition and repair of the endogenous mutagen 8-oxoguanine in DNA. *Nature* **403**, 859–866 (2000). 10706276 10.1038/35002510

[35] Faucher, F., Doublié, S. & Jia, Z. 8-Oxoguanine DNA glycosylases: one lesion, three subfamilies. *Int. J. Mol. Sci.* **13**, 6711–6729 (2012). 22837659 10.3390/ijms13066711 PMC3397491

[36] Shigdel, U. K. et al. The trajectory of intrahelical lesion recognition and extrusion by the human 8-oxoguanine DNA glycosylase. *Nat. Commun.* **11**, 4437 (2020). 10.1038/s41467-020-18290-2 PMC7477556 32895378

[37] Qi, Y. et al. Encounter and extrusion of an intrahelical lesion by a DNA repair enzyme. *Nature* **462**, 762–766 (2009). 20010681 10.1038/nature08561 PMC2951314

[38] Fromme, J. C., Banerjee, A., Huang, S. J. & Verdine, G. L. Structural basis for removal of adenine mispaired with 8-oxoguanine by MutY adenine DNA glycosylase. *Nature* **427**, 652–656 (2004). 14961129 10.1038/nature02306

[39] Gardiner-Garden, M. & Frommer, M. CpG islands in vertebrate genomes. *J. Mol. Biol.* **196**, 261–282 (1987). 3656447 10.1016/0022-2836(87)90689-9

[40] Larsen, F., Gundersen, G., Lopez, R. & Prydz, H. CpG islands as gene markers in the human genome. *Genomics* **13**, 1095–1107 (1992). 1505946 10.1016/0888-7543(92)90024-m

[41] Takai, D. & Jones, P. A. Comprehensive analysis of CpG islands in human chromosomes 21 and 22. *Proc. Natl Acad. Sci. USA* **99**, 3740–3745 (2002). 11891299 10.1073/pnas.052410099 PMC122594

[42] Deaton, A. M. & Bird, A. CpG islands and the regulation of transcription. *Genes Dev.* **25**, 1010–1022 (2011). 21576262 10.1101/gad.2037511 PMC3093116

[43] Baylin, S. B. DNA methylation and gene silencing in cancer. *Nat. Clin. Pr. Oncol.* **2**, S4–S11 (2005). 10.1038/ncponc0354 16341240

[44] Suzuki, M. M. & Bird, A. DNA methylation landscapes: provocative insights from epigenomics. *Nat. Rev. Genet.* **9**, 465–476 (2008). 18463664 10.1038/nrg2341

[45] Schubeler, D. Function and information content of DNA methylation. *Nature* **517**, 321–326 (2015). 25592537 10.1038/nature14192

[46] Kasymov, R. D. et al. Excision of 8-oxoguanine from methylated CpG dinucleotides by human 8-oxoguanine DNA glycosylase. *FEBS Lett.* **587**, 3129–3134 (2013). 23954288 10.1016/j.febslet.2013.08.008

[47] Bilotti, K., Kennedy, E. E., Li, C. & Delaney, S. Human OGG1 activity in nucleosomes is facilitated by transient unwrapping of DNA and is influenced by the local histone environment. *DNA Repair* **59**, 1–8 (2017). 28892740 10.1016/j.dnarep.2017.08.010 PMC5643252

[48] Odell, I. D., Wallace, S. S. & Pederson, D. S. Rules of engagement for base excision repair in chromatin. *J. Cell Physiol.* **228**, 258–266 (2013). 22718094 10.1002/jcp.24134 PMC3468691

[49] Luger, K., Mäder, A. W., Richmond, R. K., Sargent, D. F. & Richmond, T. J. Crystal structure of the nucleosome core particle at 2.8 A resolution. *Nature* **389**, 251–260 (1997). 9305837 10.1038/38444

[50] Cutter, A. R. & Hayes, J. J. A brief review of nucleosome structure. *FEBS Lett.* **589**, 2914–2922 (2015). 25980611 10.1016/j.febslet.2015.05.016 PMC4598263

[51] Kobayashi, W. & Kurumizaka, H. Structural transition of the nucleosome during chromatin remodeling and transcription. *Curr. Opin. Struct. Biol.* **59**, 107–114 (2019). 31473439 10.1016/j.sbi.2019.07.011

[52] Olmon, E. D. & Delaney, S. Differential ability of five DNA glycosylases to recognize and repair damage on nucleosomal DNA. *ACS Chem. Biol.* **12**, 692–701 (2017). 28085251 10.1021/acschembio.6b00921 PMC6557264

[53] Menoni, H. et al. ATP-dependent chromatin remodeling is required for base excision repair in conventional but not in variant H2A.Bbd nucleosomes. *Mol. Cell Biol.* **27**, 5949–5956 (2007). 17591702 10.1128/MCB.00376-07 PMC1952146

[54] Menoni, H., Shukla, M. S., Gerson, V., Dimitrov, S. & Angelov, D. Base excision repair of 8-oxoG in dinucleosomes. *Nucleic Acids Res.* **40**, 692–700 (2012). 21930508 10.1093/nar/gkr761 PMC3258150

[55] Popov, A. V. et al. Molecular dynamics approach to identification of new OGG1 cancer-associated somatic variants with impaired activity. *J. Biol. Chem.* **296**, 100229 (2021). 33361155 10.1074/jbc.RA120.014455 PMC7948927

[56] Fromme, J. C., Bruner, S. D., Yang, W., Karplus, M. & Verdine, G. L. Product-assisted catalysis in base-excision DNA repair. *Nat. Struct. Biol.* **10**, 204–211 (2003). 12592398 10.1038/nsb902

[57] Radom, C. T., Banerjee, A. & Verdine, G. L. Structural characterization of human 8-oxoguanine DNA glycosylase variants bearing active site mutations. *J. Biol. Chem.* **282**, 9182–9194 (2007). 17114185 10.1074/jbc.M608989200

[58] Singh, K. K., Sigala, B., Sikder, H. A. & Schwimmer, C. Inactivation of *Saccharomyces cerevisiae* OGG1 DNA repair gene leads to an increased frequency of mitochondrial mutants. *Nucleic Acids Res.* **29**, 1381–1388 (2001). 11239005 10.1093/nar/29.6.1381 PMC29743

[59] O’Hagan, H. M. et al. Oxidative damage targets complexes containing DNA methyltransferases, SIRT1, and polycomb members to promoter CpG Islands. *Cancer Cell* **20**, 606–619 (2011). 22094255 10.1016/j.ccr.2011.09.012 PMC3220885

[60] Cai, Y. et al. The NuRD complex cooperates with DNMTs to maintain silencing of key colorectal tumor suppressor genes. *Oncogene* **33**, 2157–2168 (2014). 23708667 10.1038/onc.2013.178 PMC3883927

[61] Nash, H. M., Lu, R., Lane, W. S. & Verdine, G. L. The critical active-site amine of the human 8-oxoguanine DNA glycosylase, hOgg1: direct identification, ablation and chemical reconstitution. *Chem. Biol.* **4**, 693–702 (1997). 9331411 10.1016/s1074-5521(97)90225-8

[62] Jumper, J. et al. Highly accurate protein structure prediction with AlphaFold. *Nature* **596**, 583–589 (2021). 34265844 10.1038/s41586-021-03819-2 PMC8371605

[63] Yuan, Q. et al. AlphaFold2-aware protein-DNA binding site prediction using graph transformer. *Brief Bioinform.* **23**, bbab564 (2022). 10.1093/bib/bbab564 35039821

[64] D’Augustin, O. et al. Identification of key residues of the DNA glycosylase OGG1 controlling efficient DNA sampling and recruitment to oxidized bases in living cells. *Nucleic Acids Res.* **51**, 4942–4958 (2023). 37021552 10.1093/nar/gkad243 PMC10250219

[65] Hashiguchi, K., Stuart, J. A., de Souza-Pinto, N. C. & Bohr, V. A. The C-terminal alphaO helix of human Ogg1 is essential for 8-oxoguanine DNA glycosylase activity: the mitochondrial beta-Ogg1 lacks this domain and does not have glycosylase activity. *Nucleic Acids Res.* **32**, 5596–5608 (2004). 15494448 10.1093/nar/gkh863 PMC524278

[66] D’Augustin, O., Huet, S., Campalans, A. & Radicella, J. P. Lost in the crowd: how does human 8-oxoguanine DNA glycosylase 1 (OGG1) find 8-oxoguanine in the genome? *Int. J. Mol. Sci.* **21**, 8360 (2020). 10.3390/ijms21218360 PMC7664663 33171795

[67] Blainey, P. C., van Oijen, A. M., Banerjee, A., Verdine, G. L. & Xie, X. S. A base-excision DNA-repair protein finds intrahelical lesion bases by fast sliding in contact with DNA. *Proc. Natl Acad. Sci. USA* **103**, 5752–5757 (2006). 16585517 10.1073/pnas.0509723103 PMC1458645

[68] Bilokapic, S., Strauss, M. & Halic, M. Structural rearrangements of the histone octamer translocate DNA. *Nat. Commun.* **9**, 1330 (2018). 29626188 10.1038/s41467-018-03677-z PMC5889399

[69] Zivanov, J. et al. New tools for automated high-resolution cryo-EM structure determination in RELION-3. *Elife* **7**, e42166 (2018). 10.7554/eLife.42166 PMC6250425 30412051

[70] Zhong, E. D., Bepler, T., Berger, B. & Davis, J. H. CryoDRGN: reconstruction of heterogeneous cryo-EM structures using neural networks. *Nat. Methods* **18**, 176–185 (2021). 33542510 10.1038/s41592-020-01049-4 PMC8183613

[71] Kulikova, E. et al. Visualization of complex DNA damage along accelerated ions tracks. *EPJ Web Conf.* **177**, 06002 (2018).

[72] Ondovcik, S. L., Tamblyn, L., McPherson, J. P. & Wells, P. G. Oxoguanine glycosylase 1 (OGG1) protects cells from DNA double-strand break damage following methylmercury (MeHg) exposure. *Toxicol. Sci.* **128**, 272–283 (2012). 22523232 10.1093/toxsci/kfs138

[73] Zheng, L., Tsai, B. & Gao, N. Structural and mechanistic insights into the DNA glycosylase AAG-mediated base excision in nucleosome. *Cell Discov.* **9**, 62 (2023). 37339965 10.1038/s41421-023-00560-0 PMC10281986

[74] Banerjee, A., Yang, W., Karplus, M. & Verdine, G. L. Structure of a repair enzyme interrogating undamaged DNA elucidates recognition of damaged DNA. *Nature* **434**, 612–618 (2005). 15800616 10.1038/nature03458

[75] Dyer, P. N. et al. Reconstitution of nucleosome core particles from recombinant histones and DNA in *Methods in Enzymology*, Vol. 375, 23–44 (Academic Press, 2003). 10.1016/s0076-6879(03)75002-2 14870657

[76] Shim, Y., Duan, M. R., Chen, X., Smerdon, M. J. & Min, J. H. Polycistronic coexpression and nondenaturing purification of histone octamers. *Anal. Biochem.* **427**, 190–192 (2012). 22617796 10.1016/j.ab.2012.05.006 PMC3412673

[77] Maskell, D. P. et al. Structural basis for retroviral integration into nucleosomes. *Nature* **523**, 366–369 (2015). 26061770 10.1038/nature14495 PMC4530500

[78] Farnung, L., Vos, S. M., Wigge, C. & Cramer, P. Nucleosome–Chd1 structure and implications for chromatin remodelling. *Nature* **550**, 539–542 (2017). 29019976 10.1038/nature24046 PMC5697743

[79] Mastronarde, D. N. Advanced data acquisition from electron microscopes with SerialEM. *Microsc. Microanal.* **24**, 864–865 (2018).

[80] Mindell, J. A. & Grigorieff, N. Accurate determination of local defocus and specimen tilt in electron microscopy. *J. Struct. Biol.* **142**, 334–347 (2003). 12781660 10.1016/s1047-8477(03)00069-8

[81] Zheng, S. Q. et al. MotionCor2: anisotropic correction of beam-induced motion for improved cryo-electron microscopy. *Nat. Methods* **14**, 331–332 (2017). 28250466 10.1038/nmeth.4193 PMC5494038

[82] Punjani, A., Rubinstein, J. L., Fleet, D. J. & Brubaker, M. A. cryoSPARC: algorithms for rapid unsupervised cryo-EM structure determination. *Nat. Methods* **14**, 290–296 (2017). 28165473 10.1038/nmeth.4169

[83] Emsley, P., Lohkamp, B., Scott, W. G. & Cowtan, K. Features and development of Coot. *Acta Crystallogr. D. Biol. Crystallogr.* **66**, 486–501 (2010). 20383002 10.1107/S0907444910007493 PMC2852313

[84] Pettersen, E. F. et al. UCSF Chimera-a visualization system for exploratory research and analysis. *J. Comput. Chem.* **25**, 1605–1612 (2004). 15264254 10.1002/jcc.20084

[85] Adams, P. D. et al. PHENIX: a comprehensive Python-based system for macromolecular structure solution. *Acta Crystallogr. D. Biol. Crystallogr.* **66**, 213–221 (2010). 20124702 10.1107/S0907444909052925 PMC2815670

[86] Pettersen, E. F. et al. UCSF ChimeraX: structure visualization for researchers, educators, and developers. *Protein Sci.* **30**, 70–82 (2021). 32881101 10.1002/pro.3943 PMC7737788

