# Supplementary material for: Human 8-oxoguanine glycosylase OGG1 binds nucleosome at the dsDNA ends and the super-helical locations
Source: Commun Biol. 2024 Sep 28;7:1202. doi: 10.1038/s42003-024-06919-7 (PMC11438860; doi:10.1038/s42003-024-06919-7)
Supplement: Supplementary file 1 — Supplementary information [file 42003_2024_6919_MOESM1_ESM.pdf]

Supplemental Table 1. Cryo-EM data collection, refinement, and validations

	**OGG1–DNA (8-oxoG)** (PDB 8VX4, EMDB-43607)	**NCP with 8-oxoG at SHL-5** (PDB 8VX5, EMDB-43068)	**NCP with OGG1 at entry site** (PDB 8VX6, EMDB-43069)	**NCP with OGG1 at entry/exit and SHL-5 sites** (EMDB-430610)	**NCP with OGG1 at entry/exit and SHL+6 sites** (EMDB-430611)
**Data collection and processing**					
Magnification (k)	130	130	130	130	130
Voltage (kV)	300	300	300	300	300
Electron exposure ($e^{-}/Å^{2}$)	60	60	60	60	60
Defocus range (-μm)	1.0-2.0	1.0-2.0	1.0-2.0	1.0-2.0	1.0-2.0
Pixel size (Å)	0.828	0.828	0.828	0.828	0.828
Symmetry imposed	C1	C1	C1	C1	C1
Micrograph #	11614	4354	17124	17124	17124
Initial particle #	16,947,612	2,699,710	14,501,354	14,501,354	14,501,354
Final particle #	787,683	430,285	439,090	51,710	155,394
Map resolution (Å)	3.6	3.3	3.2	5.7	7.6
FSC threshold	0.143	0.143	0.143	0.143	0.143
**Refinement**					
Map sharpening *B* factor ($Å^2$)	-270	-142	-119		
Model composition					
Non-hydrogen atoms	3651	12564	15082		
Protein residues	311	774	1086		
Nucleotide	60	312	317		
B factors ($Å^2$)					
Protein	107.74	61.21	110.79		
Nucleotide	164.58	108.50	83.43		
R.m.s. deviations					
Bond lengths (Å)	0.004	0.004	0.004		
Bond angles (°)	0.714	0.692	0.547		
Validation					
MolProbity score	1.91	1.11	1.32		
Clashscore	11.30	3.19	5.89		
Poor rotamers (%)	0.8	0	0.9		
Ramachandran plot					
Favored (%)	95.11	98.28	98.41		
Allowed (%)	4.89	1.72	1.59		
Disallowed (%)	0	0	0		

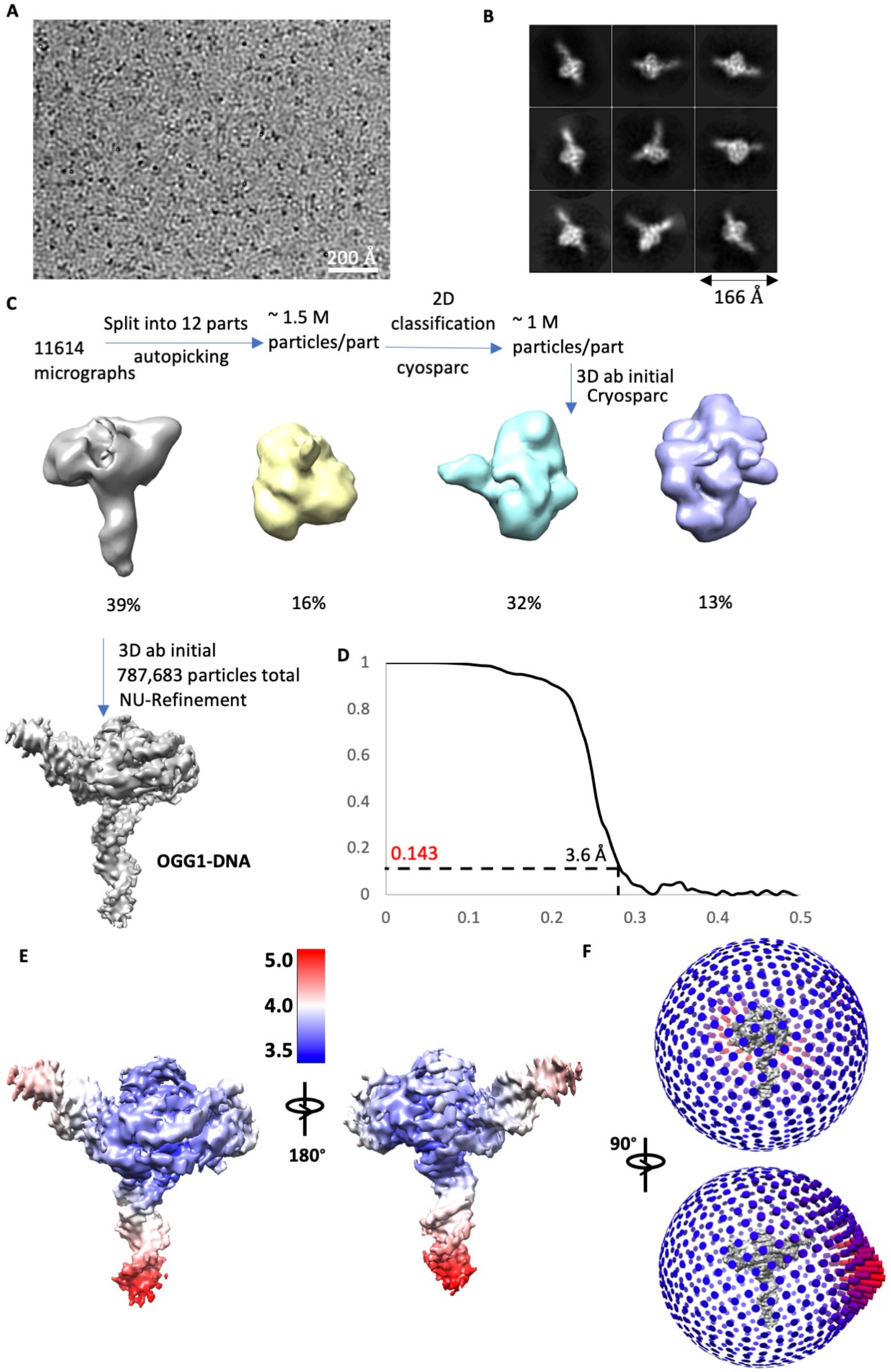

Supplementary Figure 1. Cryo-EM data processing and resolution estimation of the human OGG1–DNA complex. A) A representative electron micrograph. **B**) Selected reference-free 2D class averages. **C**) Cryo-EM data processing procedure. **D**) Gold-standard Fourier shell correlation indicates an overall resolution of 3.6 Å. **E**) Color-coded local resolution map of the final 3D map. **F**) Angular distribution of raw particles used in the final 3D reconstruction of the EM map.

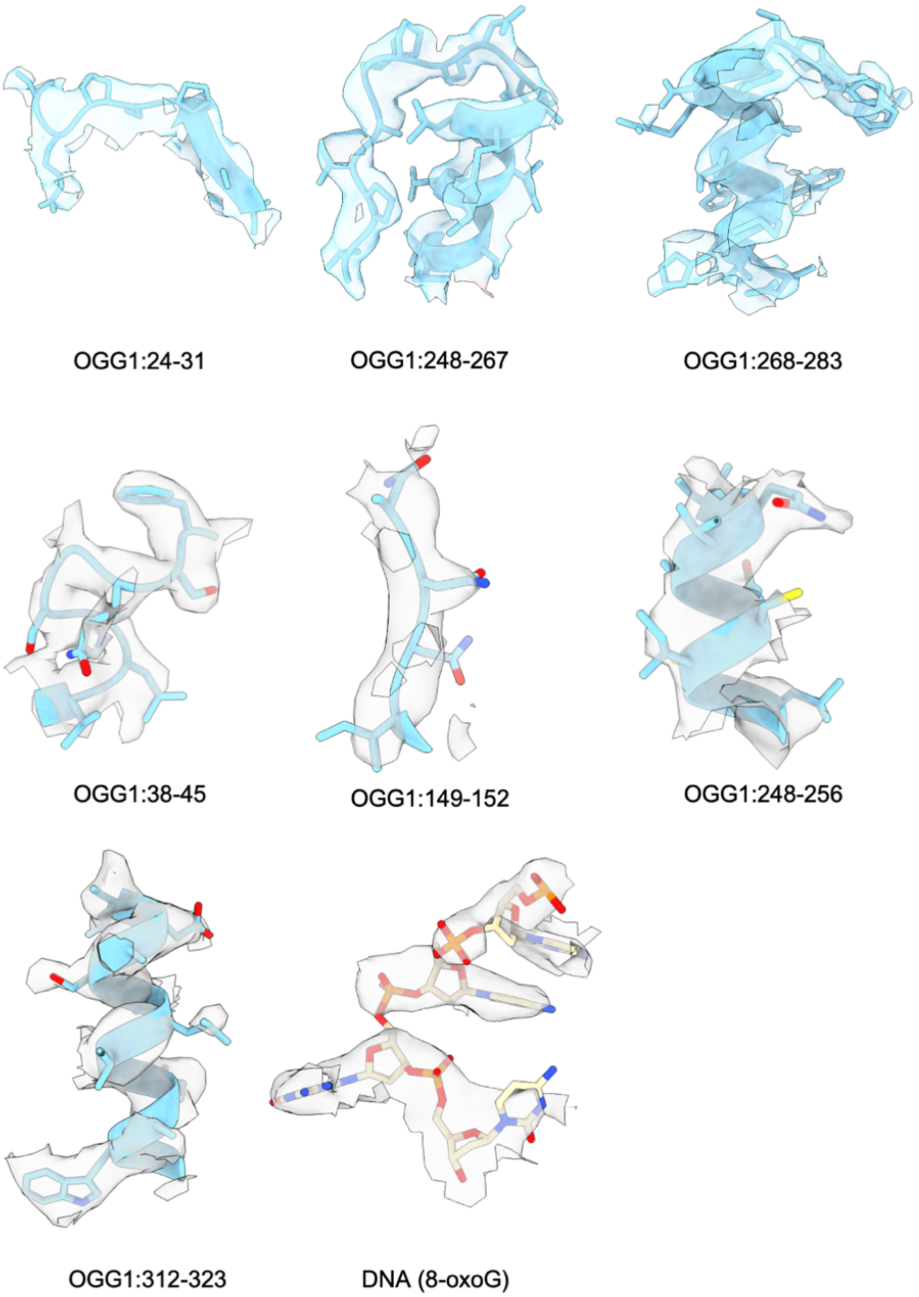

Supplementary Figure 2. Selected local EM densities. Eight OGG1 protein regions and one DNA region are superimposed with the atomic model to demonstrate the quality and fit to the EM map.

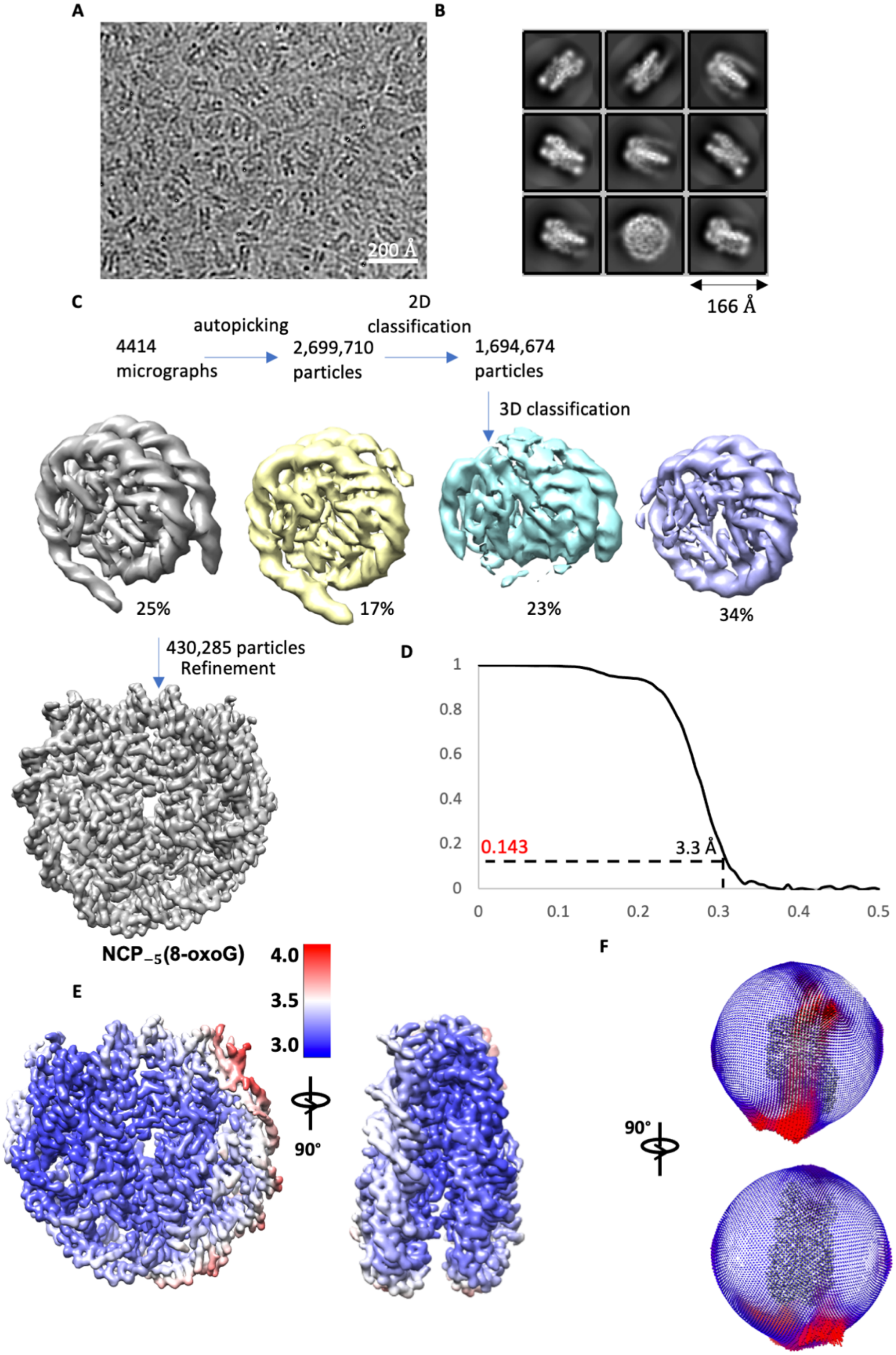

Supplementary Figure 3. Cryo-EM data processing and resolution estimation of the nucleosome with an 8-oxo-G at the SHL-5 site. A) A representative electron micrograph. **B**) Selected reference-free 2D class averages. **C**) Cryo-EM data processing procedure. **D**) Gold-standard Fourier shell correlation indicates an overall resolution of 3.3 Å. **E**) Color-coded local resolution map of the final 3D map. **F**) Angular distribution of raw particles used in the final 3D reconstruction of the EM map.

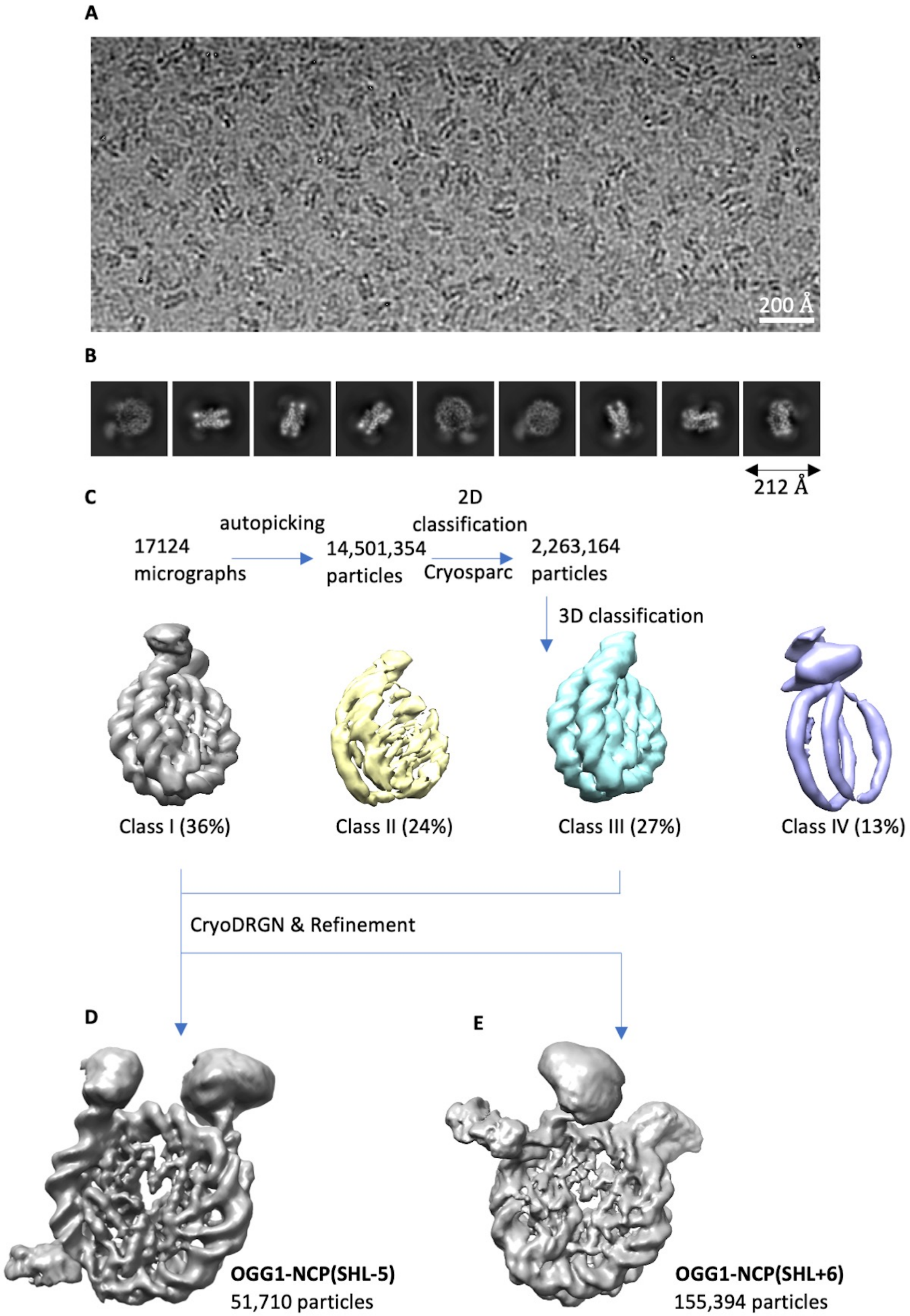

Supplementary Figure 4. Cryo-EM data processing of OGG1 bound at the nucleosome entry/exit and SHL-5, SHL+6 sites. A) A representative electron micrograph. **B**) Selected reference-free 2D class averages. **C**) Cryo-EM data processing procedure. **D-E**) The OGG1 particles bound at the NCP SHL-5 (D) and SHL+6 sites (E) were selected by CryoDRGN, as detailed in Supplementary Figure 5.

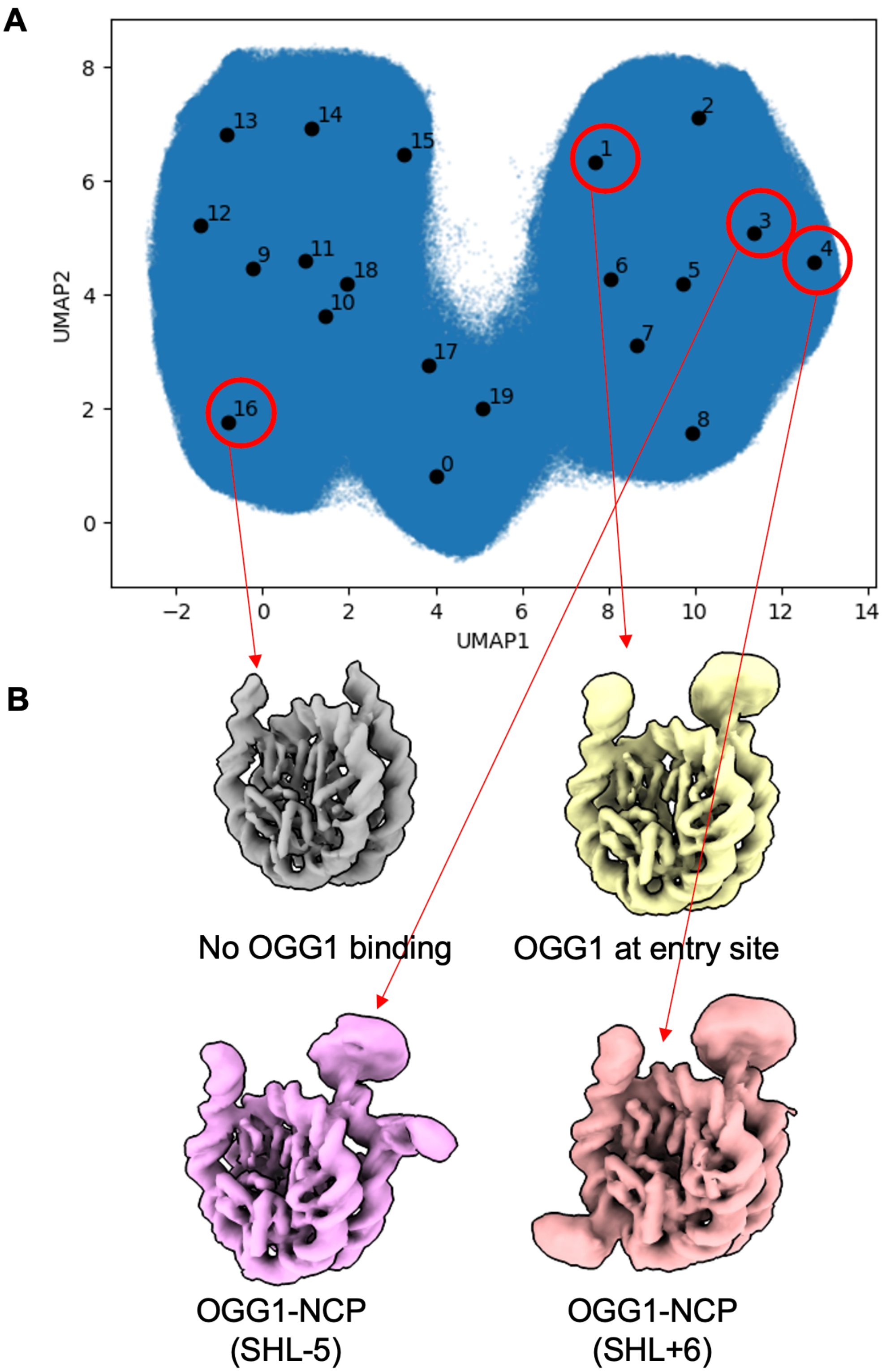

Supplementary Figure 5. Using cryoDRGN to discern different OGG1 binding states on NCP. The UMAP plot visualize the heterogeneity of the dataset (**A**). 20 cluster centers in two major regions (left and right) can be discerned. The clusters in the left region show only nucleosome densities, and a representative cluster center (#16) is shown on at the top left panel of (**B**). The clusters in the right region show clear OGG1 binding. Most of the OGG1 densities (#1) were at the entry site (top right panel, **B**), while additional OGG1 densities (#3 and #4) were observed on the NCP SHL-5 and SHL+6 sites (two lower panels, **B**).

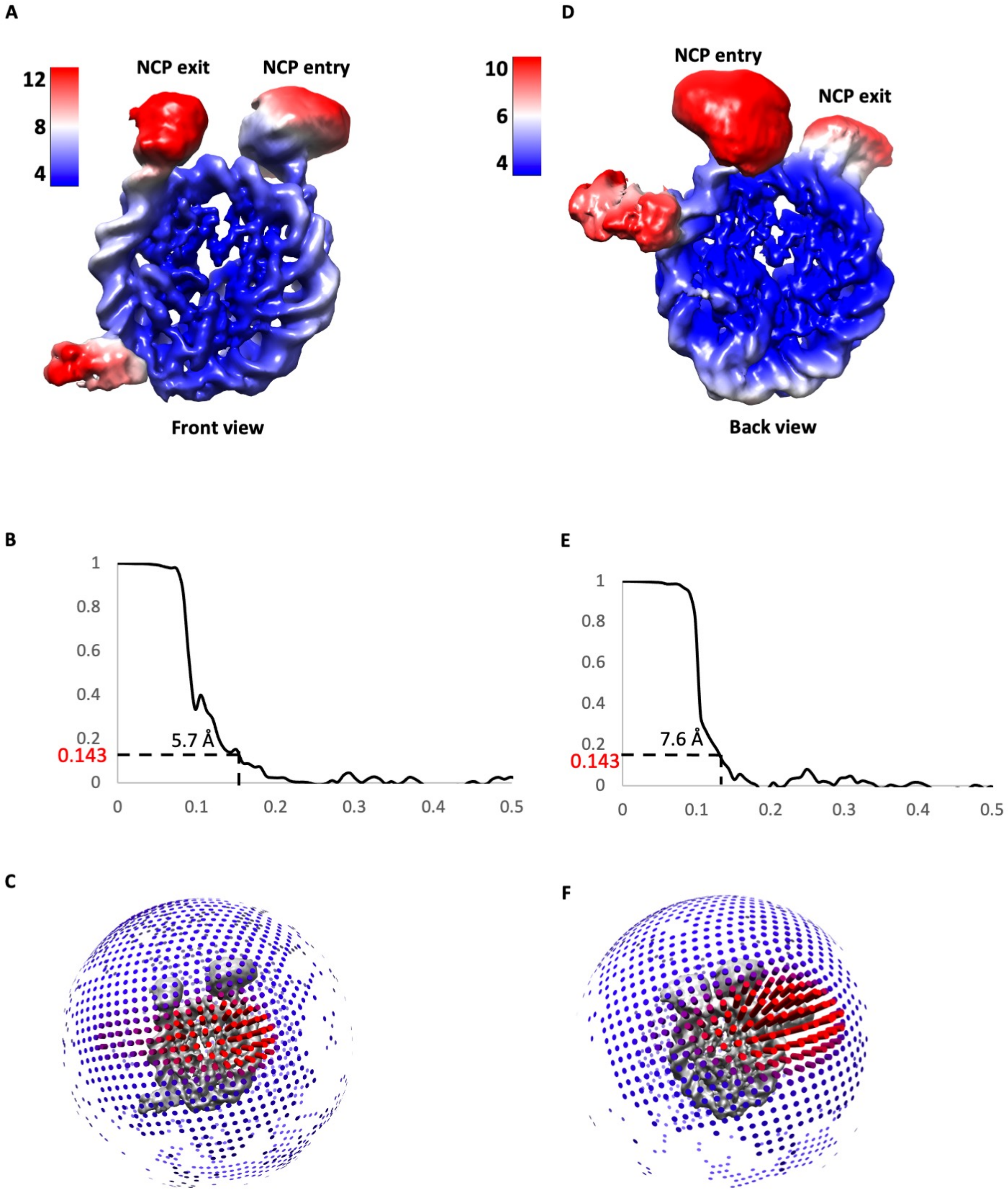

Supplementary Figure 6. Cryo-EM resolution estimation of OGG1 bound to nucleosome at entry/exit and SHL-5, SHL+6 sites. A) Color-coded local resolution map of the final 3D map of OGG1 bound at NCP (SHL-5). **B**) Gold-standard Fourier shell correlation of the two OGG1-NCP (SHL-5) half maps indicates an overall resolution of 5.7 Å. **C**) Angular distribution of raw particles used in the reconstruction of the final OGG1-NCP (SHL-5) map. **D** Color-coded local resolution map of the final 3D map of OGG1 bound at NCP (SHL+6). **E**) Gold-standard Fourier shell correlation of the two OGG1-NCP (SHL+6) half maps indicates an overall resolution of 7.6 Å. **F**) Angular distribution of raw particles used in the reconstruction of the final OGG1-NCP (SHL+6) map.

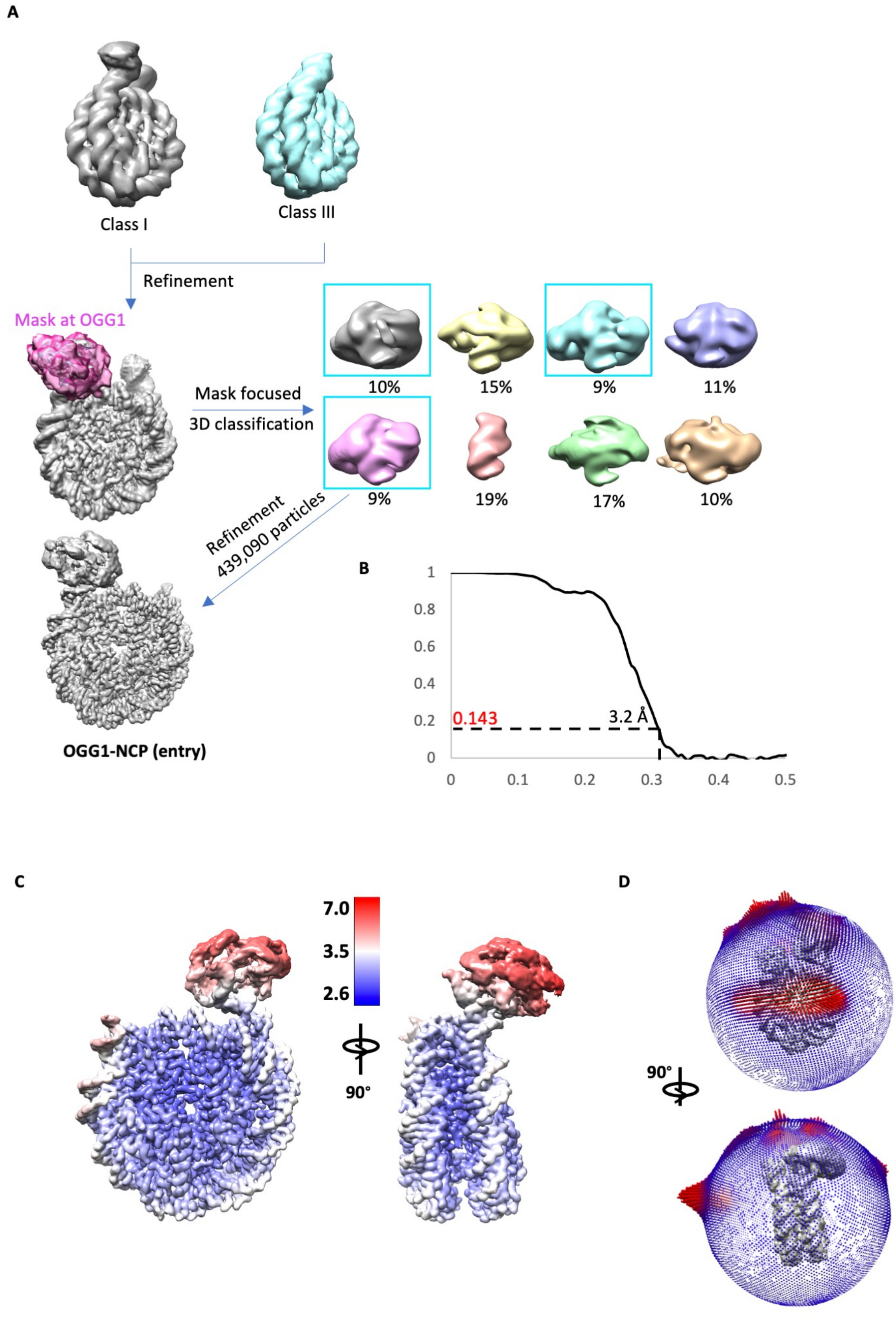

Supplementary Figure 7. Cryo-EM data processing and resolution estimation of OGG1 bound at the nucleosome entry site. A) Cryo-EM data processing procedure. **B**) Gold-standard Fourier shell correlation of the two half maps indicates an overall resolution of 3.2 Å. **C**) Color-coded local resolution map of the final 3D map. **D**) Angular distribution of particles used in reconstructing the final 3D map.

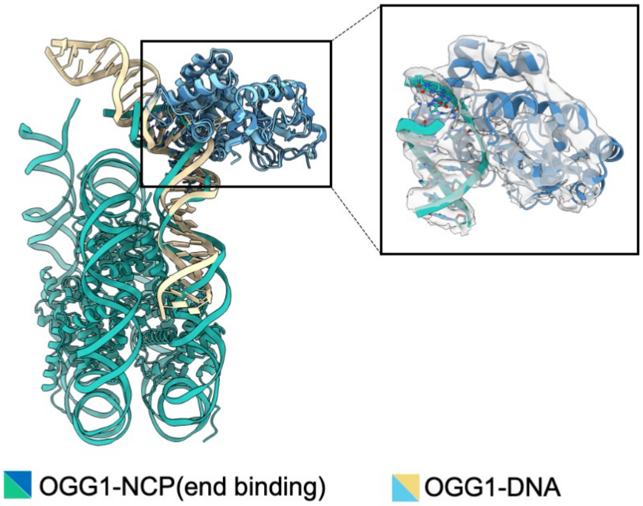

Supplementary Figure 8. Superimposition of the structure of nucleosome bound by OGG1 at the entry site with the structure of OGG1 bound to the 35-bp naked DNA. The enlarged region shows that OGG1 bends the local DNA near the end of the nucleosome DNA.

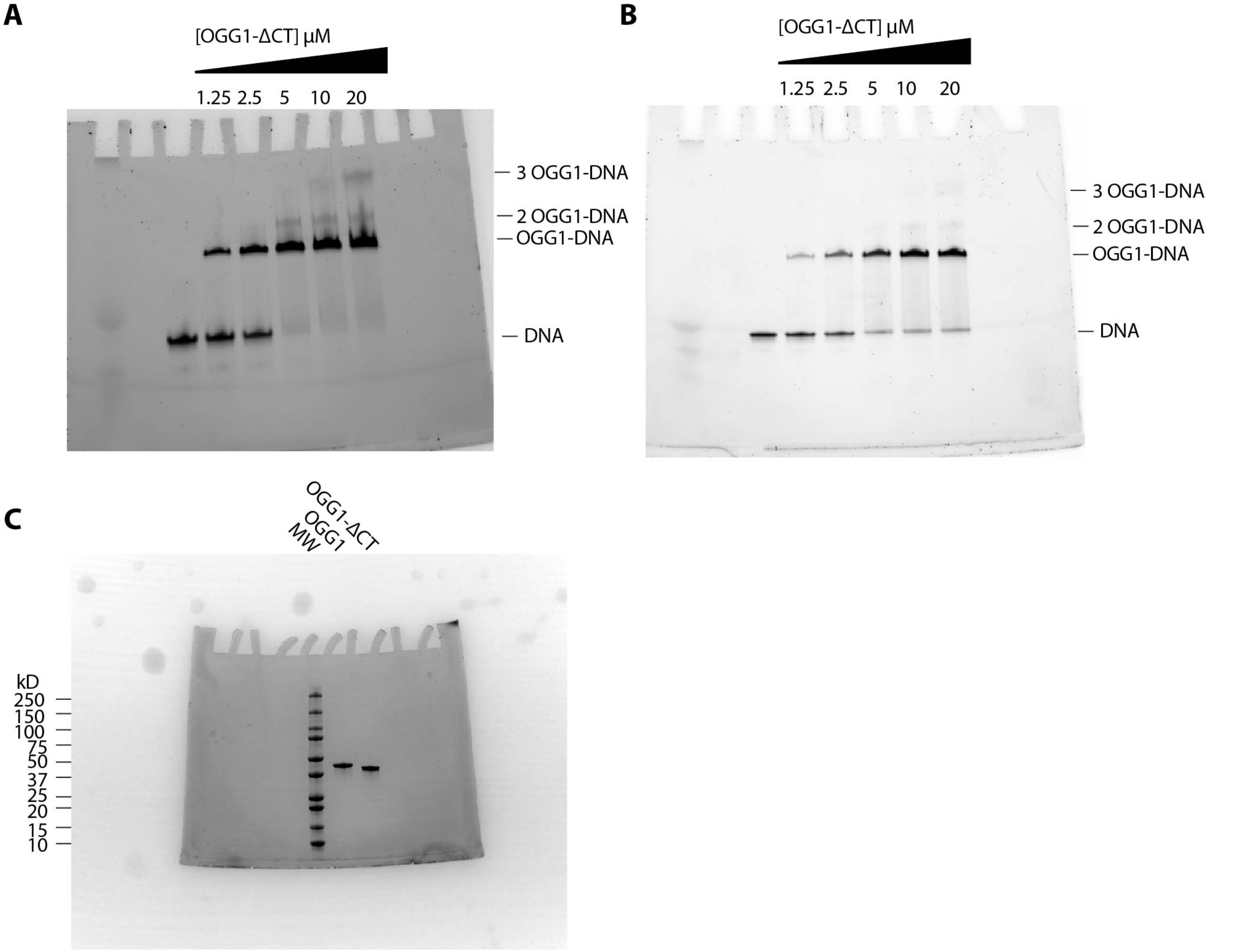

Supplementary Figure 9. **Uncropped and unedited gel images used in this study. A**) An 8-oxoG containing DNA duplex was shifted by full length OGG1 in this EMSA assay. Related to Fig. 3a. **B**) An 8-oxoG containing DNA duplex was shifted by C-terminus truncated OGG1 (OGG1-ΔCT) in the EMSA assay. Related to Fig. 3b. **C**) SDS-PAGE gel of purified OGG1 and OGG1-ΔCT used in the EMSA assay. Related to Fig. 3c.
