# Supplementary material for: Human 8-oxoguanine glycosylase OGG1 binds nucleosome at the dsDNA ends and the super-helical locations
Source: Commun Biol. 2024 Sep 28;7:1202. doi: 10.1038/s42003-024-06919-7 (PMC11438860; doi:10.1038/s42003-024-06919-7)
Supplement: Supplementary file 2 — Reporting summary [file 42003_2024_6919_MOESM2_ESM.pdf]

nature portfolio

Corresponding author(s): Huilin Li

Last updated by author(s): Sep 12, 2024

Reporting Summary

Nature Portfolio wishes to improve the reproducibility of the work that we publish. This form provides structure for consistency and transparency in reporting. For further information on Nature Portfolio policies, see our Editorial Policies and the Editorial Policy Checklist.

Statistics

For all statistical analyses, confirm that the following items are present in the figure legend, table legend, main text, or Methods section.

n/a	Confirmed	
☒	☐	The exact sample size (n) for each experimental group/condition, given as a discrete number and unit of measurement
☒	☐	A statement on whether measurements were taken from distinct samples or whether the same sample was measured repeatedly
☒	☐	The statistical test(s) used AND whether they are one- or two-sided<br>*Only common tests should be described solely by name; describe more complex techniques in the Methods section.*
☒	☐	A description of all covariates tested
☒	☐	A description of any assumptions or corrections, such as tests of normality and adjustment for multiple comparisons
☒	☐	A full description of the statistical parameters including central tendency (e.g. means) or other basic estimates (e.g. regression coefficient) AND variation (e.g. standard deviation) or associated estimates of uncertainty (e.g. confidence intervals)
☒	☐	For null hypothesis testing, the test statistic (e.g. F, t, r) with confidence intervals, effect sizes, degrees of freedom and P value noted<br>*Give P values as exact values whenever suitable.*
☒	☐	For Bayesian analysis, information on the choice of priors and Markov chain Monte Carlo settings
☒	☐	For hierarchical and complex designs, identification of the appropriate level for tests and full reporting of outcomes
☒	☐	Estimates of effect sizes (e.g. Cohen's d, Pearson's r), indicating how they were calculated

Our web collection on statistics for biologists contains articles on many of the points above.

Software and code

Policy information about availability of computer code

Data collection	The Cryo-EM data was collected using SerialEM version 4.0.0 beta.
Data analysis	The software for structure determination and illustration includes Relion-5.0, MotionCorr2.1.3.2, CTFFIND4.1.10,CryoSPARC-v4.3.1, Coot-0.9.2, UCSF ChimeraX-1.4, UCSF Pyem-v0.5, Phenix1.2.1-5207-000, CryoDRGN-v0.3.2. DNA binding sites was predicted by web server GraphSite:https://bio-web1.nscc-gz.cn/app/graphsite. DNA bending angle was measured using a script in GitHub (https://github.com/Sunyp-IM/DNA-bending-angle).

For manuscripts utilizing custom algorithms or software that are central to the research but not yet described in published literature, software must be made available to editors and reviewers. We strongly encourage code deposition in a community repository (e.g. GitHub). See the Nature Portfolio guidelines for submitting code & software for further information.

Data

Policy information about availability of data

All manuscripts must include a data availability statement. This statement should provide the following information, where applicable:

- Accession codes, unique identifiers, or web links for publicly available datasets
- A description of any restrictions on data availability
- For clinical datasets or third party data, please ensure that the statement adheres to our policy

Atomic coordinates and Cryo-EM maps reported in this study have been deposited in the Protein Data Bank (http://www.rcsb.org) and the Electron Microscopy

Data Bank (https://ebi.ac.uk/pdbe/emdb/) as follows;
PDB ID 8VX4, EMD-43607: Human OGG1 binding at 8-oxoG of a 35 bp DNA duplex
PDB ID 8VX5, EMD-43608: Nucleosome core particle containing 8-oxoG DNA damage
PDB ID 8VX6, EMD-43609: Human OGG1 binds nucleosome at the entry site
EMD-43610: Human OGG1 binds nucleosome at SHL(-5)
EMD-43611: Human OGG1 binds nucleosome at SHL(+6)

Research involving human participants, their data, or biological material

Policy information about studies with human participants or human data. See also policy information about sex, gender (identity/presentation), and sexual orientation and race, ethnicity and racism.

Reporting on sex and gender	Human participants, their data were not involved in this study.
Reporting on race, ethnicity, or other socially relevant groupings	Human participants, their data were not involved in this study.
Population characteristics	Human participants, their data were not involved in this study.
Recruitment	Human participants, their data were not involved in this study.
Ethics oversight	Human participants, their data were not involved in this study.

Note that full information on the approval of the study protocol must also be provided in the manuscript.

Field-specific reporting

Please select the one below that is the best fit for your research. If you are not sure, read the appropriate sections before making your selection.

☒ Life sciences ☐ Behavioural & social sciences ☐ Ecological, evolutionary & environmental sciences

For a reference copy of the document with all sections, see nature.com/documents/nr-reporting-summary-flat.pdf

Life sciences study design

All studies must disclose on these points even when the disclosure is negative.

Sample size	No statistical method was used to determine sample size
Data exclusions	The micrographs with the estimated resolutions lower than 8 Å after CTF estimation were discarded. Those particles did not produce 2D class averages or 3D class maps with defined features were excluded after 2D and 3D classifications. This criteria is empirical but is a standard image processing practice in the cryo-EM community.
Replication	Each particle represents one OGG1 complex. The 3D maps of OGG1-DNA and OGG1-NCP were generated from hundreds of thousands particles.
Randomization	The resolution was estimated by the gold standard Fourier shell correlation (FSC) at the threshold of 0.143. Two independenly resconstructed half-maps from nonoverlapping particles were used to calculate the FSC curves.
Blinding	No blinding was used in this study.

Reporting for specific materials, systems and methods

We require information from authors about some types of materials, experimental systems and methods used in many studies. Here, indicate whether each material, system or method listed is relevant to your study. If you are not sure if a list item applies to your research, read the appropriate section before selecting a response.

Materials & experimental systems

n/a	Involved in the study	
☒	☐	Antibodies
☒	☐	Eukaryotic cell lines
☒	☐	Palaeontology and archaeology
☒	☐	Animals and other organisms
☒	☐	Clinical data
☒	☐	Dual use research of concern
☒	☐	Plants

Methods

n/a	Involved in the study	
☒	☐	ChIP-seq
☒	☐	Flow cytometry
☒	☐	MRI-based neuroimaging

Plants

Seed stocks	Plants or Plant Data were not involved in this study.
Novel plant genotypes	Plants or Plant Data were not involved in this study.
Authentication	Plants or Plant Data were not involved in this study.
